# Intrinsic features of Zika Virus non-structural proteins NS2A and NS4A in the regulation of viral replication

**DOI:** 10.1371/journal.pntd.0010366

**Published:** 2022-05-06

**Authors:** Yufeng Yu, Chengfeng Gao, Chunxia Wen, Peng Zou, Xian Qi, Carol J. Cardona, Zheng Xing

**Affiliations:** 1 Shanxi Provincial Key Laboratory for Functional Proteins, School of Basic Medical Sciences, Shanxi Medical University, Taiyuan, Shanxi, China; 2 Jiangsu Key Laboratory of Molecular Medicine, Medical school, Nanjing University, Nanjing, Jiangsu, China; 3 Shanghai Public Health Clinical Center, Fudan University, Shanghai, China; 4 Department of Acute Infectious Diseases Control and Prevention, Jiangsu Provincial Center for Disease Control and Prevention, Nanjing, Jiangsu, China; 5 Department of Veterinary Biomedical Sciences, College of Veterinary Medicine, University of Minnesota at Twin Cities, Saint Paul, Minnesota, United States of America; Johns Hopkins University, Bloomberg School of Public Health, UNITED STATES

## Abstract

Zika virus (ZIKV) is a mosquito-borne flavivirus and can cause neurodevelopmental disorders in fetus. As a neurotropic virus, ZIKV persistently infects neural tissues during pregnancy but the viral pathogenesis remains largely unknown. ZIKV has a positive-sense and single-stranded RNA genome, which encodes 7 non-structural (NS) proteins, participating in viral replication and dysregulation of host immunity. Like those in many other viruses, NS proteins are considered to be products evolutionarily beneficiary to viruses and some are virulence factors. However, we found that some NS proteins encoded by ZIKV genome appeared to function against the viral replication. In this report we showed that exogenously expressed ZIKV NS2A and NS4A inhibited ZIKV infection by inhibiting viral RNA replication in microglial cells and astrocytes. To understand how viral NS proteins suppressed viral replication, we analyzed the transcriptome of the microglial cells and astrocytes and found that expression of NS4A induced the upregulation of ISGs, including MX1/2, OAS1/2/3, IFITM1, IFIT1, IFI6, IFI27, ISG15 or BST2 through activating the ISGF3 signaling pathway. Upregulation of these ISGs seemed to be related to the inhibition of ZIKV replication, since the anti-ZIKV function of NS4A was partially attenuated when the cells were treated with Abrocitinib, an inhibitor of the ISGF3 signaling pathway, or were knocked down with STAT2. Aborting the protein expression of NS4A, but not its nucleic acid, eliminated the antiviral activity of NS4A effectively. Dynamic expression of viral NS proteins was examined in ZIKV-infected microglial cells and astrocytes, which showed comparatively NS4A occurred later than other NS proteins during the infection. We hypothesize that NS4A may possess intrinsic features to serve as a unique type of pathogen associated molecular pattern (PAMP), detectable by the cells to induce an innate immune response, or function with other mechanisms, to restrict the viral replication to a certain level as a negative feedback, which may help ZIKV maintain its persistent infection in fetal neural tissues.

## Author summary

The birth of microcephaly infants due to ZIKV infection in pregnant women is related to ZIKV persistent infection. However, it is unclear how ZIKV maintains its persistent infection. In this work, we observed the delayed appearance of ZIKV NS4A protein in neuroglia including microglia and astrocytes compared with other non-structural proteins. Subsequently, we revealed that ZIKV NS4A inhibited viral RNA replication by activating the ISGF3 signaling pathway and inducing the production of ISGs. Aborting NS4A protein expression totally rescued ZIKV viral replication. Our study, combined with the previous findings, suggests that viral non-structural proteins may regulate viral replication, thus perpetuating ZIKV infection. Our hypothesis provides a mechanism for ZIKV to maintain its status of a persistent infection during viral infection in fetus, which can shed lights on our further understanding of viral neuropathogenesis in ZIKV infection.

## Introduction

Zika Virus (ZIKV) belongs to the genus *flavivirus* in the family *flaviviridae*, which includes Dengue virus (DENV), Japanese encephalitis virus (JEV), and West Nile virus (WNV) [1]. The viral genome is a single-stranded RNA of ∼11 kb, encoding three structural proteins (capsid, pre-membrane and envelope) and seven non-structural (NS) proteins (NS1, NS2A, NS2B, NS3, NS4A, NS4B, NS5) [2]. The structural proteins are responsible for the formation of viral particles, while the NS proteins are involved in viral replication, organization and evasion from host immunity [3]. In general ZIKV causes asymptomatic infections [4] or mild flu-like symptoms [5].When ZIKV infects pregnant women, the infection could lead to the birth of microcephaly infants [6–8]. ZIKV also causes Guillain-Barré syndrome in some adults [9–11]. The mechanism of neurological disorders caused by ZIKV has been extensively investigated in the past years.

Microglia, the resident macrophages of the central nervous system (CNS), have a perivascular localization which allows them to monitor the influx of blood-borne components into the CNS [12]. Our and other studies show that microglia are highly susceptible to ZIKV [13]. *In vitro* culture and histology of fetal brain tissues from the brain with microcephaly have shown that ZIKV activates microglia and induces high levels of pro-inflammatory cytokines [14,15]. The inflammatory factors may be detrimental to the development of the fetal brain [16]. In addition, microglia-like cells (pMGLs) could invade and initiate neuronal infection when ZIKV infected-pMGLs were co-cultured with neurospheres [17]. Because microglia originate from primitive macrophages close to the maternal vasculature, they may act as a viral reservoir for ZIKV and help establish an infection of the fetal brain [17].

Astrocytes, the most abundant cells of the CNS, have a number of neuroprotective functions, including maintaining the integrity of the blood-brain barrier (BBB), regulating synapses function and promoting neuronal repair [18]. Viral infection of astrocytes leads to increased permeability of the BBB and the entry of neurotoxic substances into the brain. Therefore, astrocytes have a critical impact on viral neuropathogenesis [18,19]. ZIKV infects astrocytes by recognizing AXL receptors and regulates their immune response [20,21]. ZIKV can persistently infect human fetal astrocytes (HFAs) at least one month, and no obvious apoptosis is induced [22]. The above findings suggest that microglia and astrocytes may play an important role in neurological disease induced by ZIKV, but how the virus maintain its status of persistent infection in infants remains unknown.

Progress has been made in the study on ZIKV NS proteins in viral pathogenesis. ZIKV NS1, NS3, NS4B and NS5 all inhibit the interferon signaling pathway [23–26] and NS2B-NS3 impedes JAK-STAT signaling pathway [23]. NS2A and NS4A may play a role in ZIKV-induced neurological disorders. ZIKV NS2A attenuates the proliferation of radial glial cells and causes defects of adherent junction proteins in human forebrain organoids [27]. Co-expression of NS4A and NS4B in fetal neural stem cells inhibits Akt-mTOR signaling pathway, which is one of the key cellular pathways essential for brain development and autophagy regulation by promoting autophagy and inhibiting neurogenesis in human fetal neural stem cells [28]. NS4A may also inhibit brain development through inhibiting the function of Ankle2, whose mutations cause an autosomal recessive microcephaly in humans [29]. However, studies on ZIKV NS proteins are mostly in non-neural cells.

In this study, we confirmed that ZIKV could infect human microglial cells and astrocytes by examining the replication of viral genome and the expression of viral proteins. We observed a late appearance of NS4A during viral infection in human microglia and astrocytes and wondered whether this delayed appearance of NS4A had a unique role in viral neuropathogenesis. To our surprise, expression of NS4A exhibited an activity against ZIKV replication, which prompted us to analyze the underlying mechanism and possible ramification of this intrinsic “viral protein against itself” feature. We propose that ZIKV utilizes its NS4A to suppress its replication via a unique mechanism, so that viral persistent infection could be maintained and ZIKV stay in fetal neural tissues for extended presence.

## Materials and methods

### Cells and viruses

HMC3 cells were maintained in Minimum Essential Medium (MEM) (Gibco, ThermoFisher) supplemented with 10% fetal bovine serum (FBS, Gibco) at 37°C in 5% CO_2_. U251, HeLa, HEK293, Vero and BHK21 cells were maintained in Dulbecco’s modified Eagle medium (DMEM) (Gibco, ThermoFisher) with 10% FBS. ZIKV strain SZ01/2016 (GenBank number: KU866423) was isolated from a patient who returned from Samoa [30]. ZIKV strains MR766 (#VR1838) were obtained from ATCC. SFTSV (JS-2010-14 strain) was isolated from peripheral blood samples of a patient by Jiangsu Provincial Centers for Disease Control and Prevention [31]. EV-A71 (Fuyang strain) was kindly provided by Dr. Wu Bin at Jiangsu Provincial Centers for Disease Control and Prevention [32]. All viruses were propagated in Vero cells and the virus stock titers were determined by a plaque formation unit (PFU) assay.

### Construction of cell lines

To construct microglial and astrocytes cells expressing ZIKV NS proteins, the cDNA of NS1, NS2A, NS2B, NS3, NS4A and NS4B were amplified from ZIKV SZ01/2016 and cloned into pLVML-3×HA-MCS-IRES-Puro (MiaoLing Plasmid Sharing Platform, China) or pRK5-HA vector. The empty vector pLVML-3×HA-MCS-IRES-Puro or pLVML-3×HA-MCS-IRES-Puro-NS, psPAX2 and PMD-2.G were co-transfected into HEK293 cells in a 1:1:1 ratio by Vigofect (Vigorous Biotechnology, Beijing, China) and the lentiviruses in culture supernatant were collected at 48 h post transfection. Microglial and astrocytes, HMC3 and U251 cells, respectively, were infected by the lentiviruses and the infected cells were screened with 2 or 8 μg/ml puromycin (Selleck, Shanghai, China) for 48 h post infection (p.i.). Two weeks later, HMC3 and U251 cells stably expressing ZIKV NS proteins were validated by immunofluorescence assay (IFA) and western blot analysis (WB). Cell lines, which were generated using lentiviruses with empty vector, served as a control for cells expressing NS proteins. We uniformly labeled these cells as “Vector” in all figures.

To obtain cell lines expressing NS4A-X, nucleotides TAA were inserted before the start codon ATG of NS4A sequence on the pLVML-3×HA-MCS-IRES-Puro-NS4A plasmid, allowing the retention of the NS4A nucleic acid and the termination of protein expression (NS4A-X). Subsequently, cell lines stably expressing NS4A-X were obtained according to the method described above.

To achieve U251 cells knocking down STAT2, p-GIPZ-shSTAT2 and p-GIPZ were purchased from Shanghai Jiao Tong University. Subsequently, together with psPAX2 and PMD-2.G, lentiviruses were constructed and used to infect U251 cells with or without NS4A expression. The lentiviruses packaged by empty plasmid p-GIPZ were set as the negative control for knockdown of STAT2.

### Immunofluorescence assay (IFA)

To verify whether HMC3 and U251 cells stably expressed ZIKV NS protein, cells were seeded on sterile coverslips in 24-well plates at 1x10^5^ per well. 24 h later, cells were fixed with 4% paraformaldehyde (Sigma-Aldrich, St Louis, MO) for 15 min, then perforated by 0.1% Triton X-100 for 15 min, blocked by 3% BSA for 30 min. Then the cells were incubated with anti-HA rabbit antibodies (1:500, CST, USA) for 1 h at room temperature. After three PBS washes, the cells were incubated with AlexaFluor 488-labelled donkey anti-rabbit IgG (1:1,000, ThermoFisher Scientific, Wilmington, DE, USA) for 1 h at room temperature. Following five washes by PBS, the coverslips were sealed with Prolong Gold Antifade reagent with DAPI (ThermoFisher Scientific, Wilmington, DE, USA) and observed under the Olympus FLUOVIEW FV3000 confocal microscope. To detect the expression of viral dsRNA in HMC3 cells expressing NS2A or NS4A, the HMC3 cells were infected by 0.1MOI of ZIKV SZ01/2016 for 24 or 48 h. The J2 mouse monoclonal anti-dsRNA antibody (1:1000, SCICONS, Sizlaku, Hungary) was used as a primary antibody and Alexa Fluor 488-labelled donkey anti-mouse IgG (1:1000, ThermoFisher Scientific, Wilmington, DE, USA) was used as a secondary antibody.

### Western blot analysis

To test the expression of viral proteins in HMC3 or U251 cells, cells were lysed at various time points after ZIKV infection using cell lysis buffer for Western and IP (Beyotime Biotechnology, Shanghai, China) with protease inhibitors (1:100, ThermoFisher Scientific, Wilmington, DE, USA). The polyclonal anti-ZIKV E rabbit antibodies (1:1000, biodragon-immunotech, Beijing, China), anti-ZIKV NS2B, NS3, NS4A, or NS5 rabbit antibodies (1:1000, GeneTex, CA, USA), or the monoclonal anti-GAPDH antibodies (1:1000, Proteintech, Wuhan, China) were used as the primary antibodies. The horseradish peroxidase (HRP)-conjugated antibodies (1:3000) against mouse IgG or rabbit IgG obtained from Proteintech (Wuhan, China) were used as the secondary antibodies. To verify whether the expression of NS2A and NS4A activates the ISGF3 signaling pathway, HMC3 cells expressing NS2A or NS4A were collected for detection of STAT1, phospho-STAT1 (Tyr701), STAT2, phospho-STAT2 (Tyr690) and IRF9. Antibodies for the above proteins were purchased from ABclonal (Wuhan, China).

### Flow cytometry

To examine the response of HMC3 or U251 cells stably expressing ZIKV NS proteins to ZIKV infection, the above cells were infected with 0.1 MOI of ZIKV SZ01/2016. 72 or 48 h p.i. later, the cells were prepared according to the instructions of the apoptosis kit (US Everbright, Suzhou, China) as follows. Briefly, the cells were washed with PBS, then digested with 0.25% EDTA-free trypsin. After 2 washes with PBS, the cells were suspended with 100 μl binding buffer with 5 μl Annexin V and 5 μl propidium iodide (PI). After 15 min incubation at room temperature, 400 μl binding buffer was added to each sample. Finally the cells were filtered through 200 mesh gauze and immediately analyzed on a BD FACS Calibur. The collected data were further analyzed by FlowJo V10.

### Viral plaque formation unit assay

BHK21 cells were seeded on a 12-well plate at 5x10^5^ per well the day before infection. Ten-fold serially diluted viruses were inoculated to BHK21 cells in triplicates. After 2 h of infection, the virus inoculum was discarded and replaced by DMEM containing 2% FBS in 1% low melting point agarose. After the agarose was solidified at room temperature, the plates were transferred to an incubator. 4–5 days later, when viral plaques were visible, the cells were stained with crystalline violet dye in 4% paraformaldehyde (PFA) for 4 h at room temperature. The plates were washed and viral plaques were counted.

### Quantitative real time polymerase chain reaction (qRT-PCR)

To quantify viral RNA copies in cell culture medium after virus infection in HMC3 cells expressing NS2A or NS4A, the culture medium was collected at 24, 48, 72 and 96 h p.i. Viral RNA was extracted using the TIANamp Virus RNA Kit (Tiangen biotech, Beijing, China), and viral RNA copies were quantified by One Step PrimeScript RT-PCR Kit (Takara Bio, Shiga, Japan) with the QuantStudio 5 PCR instrument (ABI). The following primers were used: ZIKV-E-F-Taq (5’- GGTCAGCGTCCTCTCTAATAAACG -3’), ZIKV-E-R-Taq(5’- GCACCCTAGTGTCCACTTTTTCC-3’), Probe (5’-6-FAM-AGCCATGACCGACACCACACCGT -BHQ1-3’). Standard curves were prepared using a plasmid encoding ZIKV E gene.

To determine the expression of viral genes in cells after viral infection or the expression of NS4A-induced ISGs, total RNA was isolated from the cells using RNAiso Plus reagent (Takara Bio) and reversed by HiScript III RT SuperMix for synthesis of cDNA and qPCR analysis (+gDNA wiper) (Vazyme Biotech, Nanjing, China). Then gene expressions were quantified by TB Green Premix Ex Taq II (Tli RNaseH Plus) (Takara Bio) with the QuantStudio 5 or ABI Viia 7 PCR instrument. The primers are listed in Table 1.

**Table 1:** Sequences of primers for qRT-PCR used in the study.

Primer name	Sequence (5’ to 3’)
H-MX1-F	CACCAGCGACAAGCGGAAGTT
H-MX1-R	AGTCGTCAGTCCAGTGGCTACC
H-MX2-F	GAACAATCAGCCACCACCAGGA
H-MX2-R	TTCAGCACCAGCGGACACCT
H-OAS1-F	GCAGACGATGAGACCGACGAT
H-OAS1-R	GCACTGGCATTCAGAGGATGGT
H-OAS2-F	TGCTCTCGGTGCTTCCAACTCA
H-OAS2-R	TGGCTGCTGGCATAGAGGATGT
H-OAS3-F	ATGCCGACCTCGTGGTGTTC
H-OAS3-R	AACTGCCGCTCCTGTTGACAT
H-IFITM1-F	TCCTTCCAAGGTCCACCGTGAT
H-IFITM1-R	CGTCGCCAACCATCTTCCTGTC
H-IFIT1-F	GCGCTGGGTATGCGATCTCT
H-IFIT1-R	AAGCGGACAGCCTGCCTTAG
H-IFI6-F	GCTGGTCTGCGATCCTGAATGG
H-IFI6-R	GCTGCTGGCTACTCCTCATCCT
H-IFI27-F	AATCGCCTCGTCCTCCATAGCA
H-IFI27-R	CCTCGCAATGACAGCCGCAAT
H-BST2-F	GCAGAGGTGGAGCGACTGAGAA
H-BST2-R	AGCAGGACGGACCTTCCAAGAT
H-XAF1-F	GCCTACTTGCTGTGGTGGTCTT
H-XAF1-R	ATGTTCCTTCGACGCCTGGTT
H-ISG15-F	TGCTGGTGGTGGACAAATGCG
H-ISG15-R	CCCCTCGAAGGTCAGCCAGA
H-USP18-F	CCATCGTGCCTGGCTCACAT
H-USP18-R	AACCAACCAGGCCATGAGGG
ZIKV-E-F	GGGTTGATGTTGTCTTGGAACAT
ZIKV-E-R	AGGCTTCACCTTGTGTTGGG
ZIKV-NS3-F	TGCCATGCCACCTTCACTTCAC
ZIKV-NS3-R	CCTCGCCCATCTCAACCCTTGT
ZIKV-NS4A-F	ACAAGGGCATAGGGAAGATGGG
ZIKV-NS4A-R	AGCACCACCAGCAATAGGAACA
SFTSV-F	GCAAGATGACCAACACAGTATGGTT
SFTSV-R	CCACTAGGCCACCTAAGAGCA
EV-A71-F	CGCCCAAGGTTGTGACACGATT
EV-A71-R	ACTATGCCGACGACGCCATGTT
HA-NS4A-F	TTATGATGTCCCAGACTACGCA
HA-NS4A-R	ATTCCCAGCGAGACTGTTCC
H-β-actin-F	AAGGAGAAGCTGTGCTACGTCGC
H-β-actin-R	AGACAGCACTGTGTTGGCGTACA

### ZIKV attachment, entry, and replicon assay

ZIKV attachment and entry experiments were performed as previously described [33]. For the viral attachment assay, HMC3 cells expressing NS2A or NS4A were seeded in 6-well plates at 1x10^6^ cells per well. 24 h later, the cells were infected with ZIKV SZ01/2016 (MOI = 2) and incubated at 4°C for 1 h. The unbound viruses were removed and the cells were washed twice with PBS, followed by preparation of total RNA from the cells for measuring viral RNA copies by qRT-PCR. For the entry assay, after the incubation of the cells and virus at 4°C for 1 h, the cells were washed twice with PBS, and subsequently treated with pre-warmed MEM for 10 min at 37°C. After 3 time washes with PBS, the cells were treated with 0.25% trypsin. After another 3 time washes with PBS, the cells were collected for preparing total RNA and measuring viral RNA copies by qRT-PCR.

The replicon assay was referred to previous reports [34,35] with modifications. ZIKV replicons carrying genes encoding three structural proteins and seven NS proteins were gifted by Long Gang at Institut Pasteur of Shanghai, Chinese Academy of Sciences. Firstly, the plasmids with ZIKV replicon were linearized by Mlu1 enzyme (NEB, USA). After phenol/chloroform extraction, the linearized plasmids were transcribed into infectious viral RNA *in vitro* according to the instructions of mMESSAGE mMACHINE T7 Transcription Kit (Thermo Fisher Scientific). Subsequently, 5x10^6^ HMC3 cells expressing NS2A or NS4A were resuspended in 400 μl electrotransfer solution cytomix buffer. The mixtures of cells and 5 μg Zika RNA were transferred to sterile electroporation cuvette (4 mm, BioRad, USA). After electroporation the cells every 3 s at 450V, 25 uF, total 3 times, by Gene Pulser Xcell Electroporation System (BioRad, USA), the cells were transferred to 3.5 ml MEM containing 2% FBS immediately, and seeded in 6 cm dishes for culture at 37°C, 5% CO_2_. Finally, the cells were collected at 48 and 96 h for measuring viral RNA copies by qRT-PCR or at 24, 48, 72 and 96 h for determining the expression of NS2B by western blot assay.

### RNA-Sequencing (RNA-Seq)

The HMC3 cells expressing NS2A or NS4A were plated on 10 cm culture plates and infected with 0.1 MOI of ZIKV/SZ01. Total RNA was prepared from the cells using RNAiso Plus reagent (Takara Bio) at 24 h p.i. and sent to Gene Donovo (Guangzhou, China) for RNA-Seq.

### Statistical analysis

Statistical analyses were performed by SPSS 13.0. The differences between groups of ZIKV NS proteins and controls were evaluated by two-tailed Student’s t test and the p-value of each test was chosen according to the result of Levene’s Test for Equality of Variances. *, P < 0.05; **, P < 0.01; ***, P < 0.001.

## Results

### NS4A protein occurred later than NS2B, NS3 and NS5 during ZIKV infection

**Fig 1:**
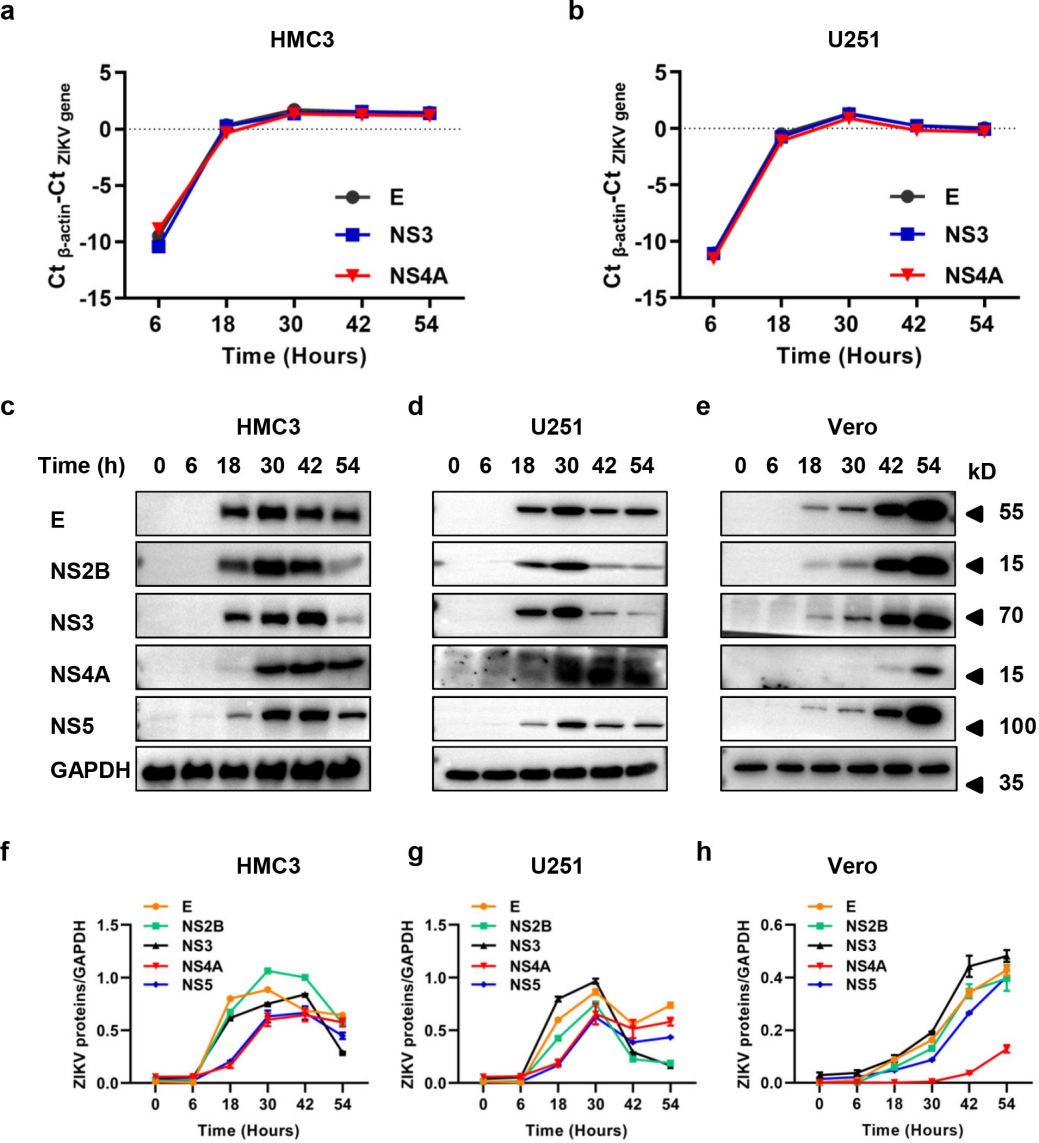
NS4A protein occurred later than NS2B, NS3 and NS5 during ZIKV infection. (a-b) Replication of ZIKV genome in microglial cells (HMC3) and astrocytic cells (U251). (c-e) Expression of ZIKV proteins in HMC3, U251 and Vero cells. (f-h) Gray scale analysis of Fig 1C (f), Fig 1D (g) and Fig 1E (h). After ZIKV/SZ01 (0.1 MOI) infection of HMC3, U251 or Vero cells for 0, 6, 18, 30, 42, and 54 h, the viral RNA or viral proteins in cell lysates was determined by qRT-PCR or western blot analysis. Data are means ± SEM of triplicate experiments. E, envelope protein; NS2B, non-structural 2B protein; NS3, non-structural 3 protein; NS4A, non-structural 4A protein; NS5, non-structural 5 protein.

ZIKV could infect human microglial and astrocytic cells and induce cytopathic effect and cell death (S1 Fig). We examined the replication of ZIKV RNA and the expression of viral proteins. ZIKV RNA replication was shown in Fig 1A (HMC3) and Fig 1B (U251) as we measured the relative viral RNA copies of genes encoding structural (E) or NS proteins (NS3 and NS4A). The RNA amplification trends of the three viral proteins were consistent, with a rapid increase before 18 h p.i. and a plateau after 30 h p.i. However, the protein expression of NS4A occurred later than other NS proteins in HMC3 and U251 cells. As shown in Fig 1C, 1D and 1F–1G, NS2B, NS3 and NS5 were highly expressed at 18, 30 or 42 h p.i, and NS4A was highly expressed at 30, 42 and 54 h p.i. It seems that the appearance of NS4A is delayed compared with other NS proteins. To test whether the delayed appearance was also present in non-neuronal cells, we examined the expression of ZIKV NS proteins in Vero cells. As shown in Fig 1E and 1H, NS4A also occurred later than NS2B, NS3 and NS5 in Vero cells.

### NS2A and NS4A were reversely associated with cell death induced by ZIKV infection

**Fig 2:**
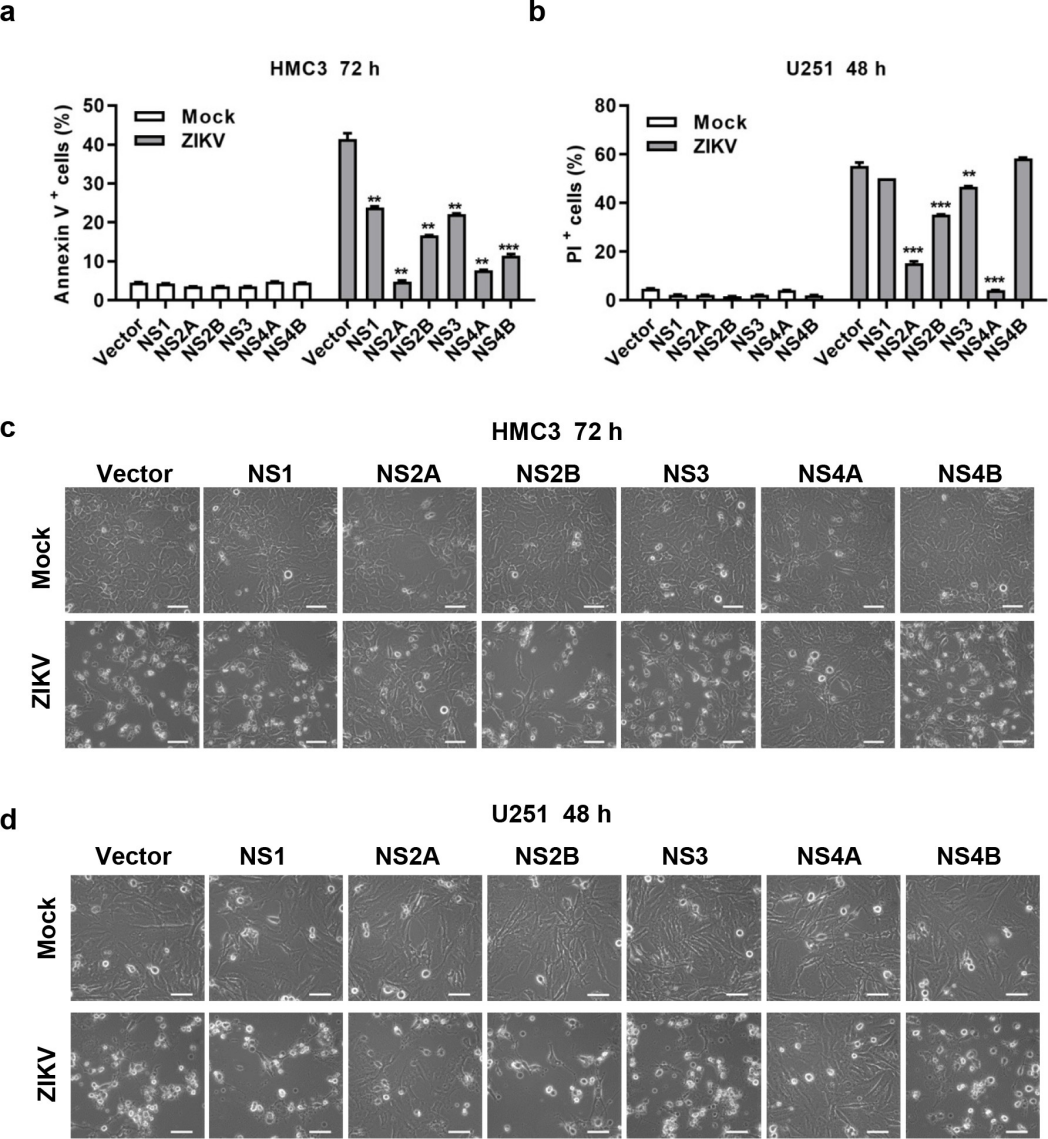
NS2A and NS4A were reversely associated with cell death induced by ZIKV infection. **(a-b)** Cell death after ZIKV/SZ01 infection of HMC3 or U251 cells expressing ZIKV NS proteins. **(c-d)** Cytopathic effect after ZIKV/SZ01 infection of HMC3 (72 h p.i.) or U251 (48 h p.i.) cells expressing ZIKV NS proteins. Scale bar, 50 μm. HMC3 or U251 cells (expressing ZIKV NS1, NS2A, NS2B, NS3, NS4A, or NS4B) were infected by 0.1 MOI of ZIKV/SZ01. Cell death was determined by flow cytometry and Annexin V/PI apoptosis kit at 48 (U251) or 72 (HMC3) h p.i. The cytopathic effect was observed under a light microscope. The differences between groups of control and NS proteins were evaluated by two-tailed Student’s t test. Data are means ± SEM of triplicate experiments; *, P< 0.05; **, P< 0.01; ***, p < 0.001.

To investigate the effect of ZIKV NS proteins on viral infection, we constructed HMC3 and U251 cell lines stably expressing ZIKV NS proteins. As shown in S2 and S3 Figs, HMC3 and U251 cells stably expressed ZIKV NS1, NS2A, NS2B, NS3, NS4A, or NS4B. Since NS5 expression inhibits glial cells growth [36], we failed to construct glial cells stably expressing NS5. HMC3 or U251 cells stably expressing ZIKV NS proteins were infected by 0.1 MOI of ZIKV for 72 or 48 h. The cell morphology changes were observed under a microscope and the cell death was detected by flow cytometry analysis after Annexin V/PI staining. As shown in Figs 2A and S4, the expression of ZIKV NS proteins significantly inhibited the number of Annexin V- positive HMC3 cells after viral infection. The expression of NS2A, NS2B, NS3, and NS4A significantly reduced the number of PI- positive U251 cells after viral infection (Figs 2B and S4). However, the morphology of HMC3 (Fig 2C) or U251 (Fig 2D) cells expressing NS1, NS2B, NS3, or NS4B was similar to the control cells after infection, and the cells were shrunk and suspended; the morphology of the infected cells expressing NS2A or NS4A remained as healthy as the control cells without infection. There were lower Annexin V or PI positive rates in cells expressing NS2A or NS4A than that of cells expressing other NS proteins, with 4.76% or 7.67% Annexin V- positive rates in HMC3 cells (Fig 2A) and 15.3% or 4.11% PI- positive rates in U251 cells (Fig 2B), respectively. NS2A and NS4A may be able to inhibit viral infection more effectively than other NS proteins, thus reducing cell death, which is what we intended to investigate.

### NS2A and NS4A inhibited ZIKV infection

**Fig 3:**
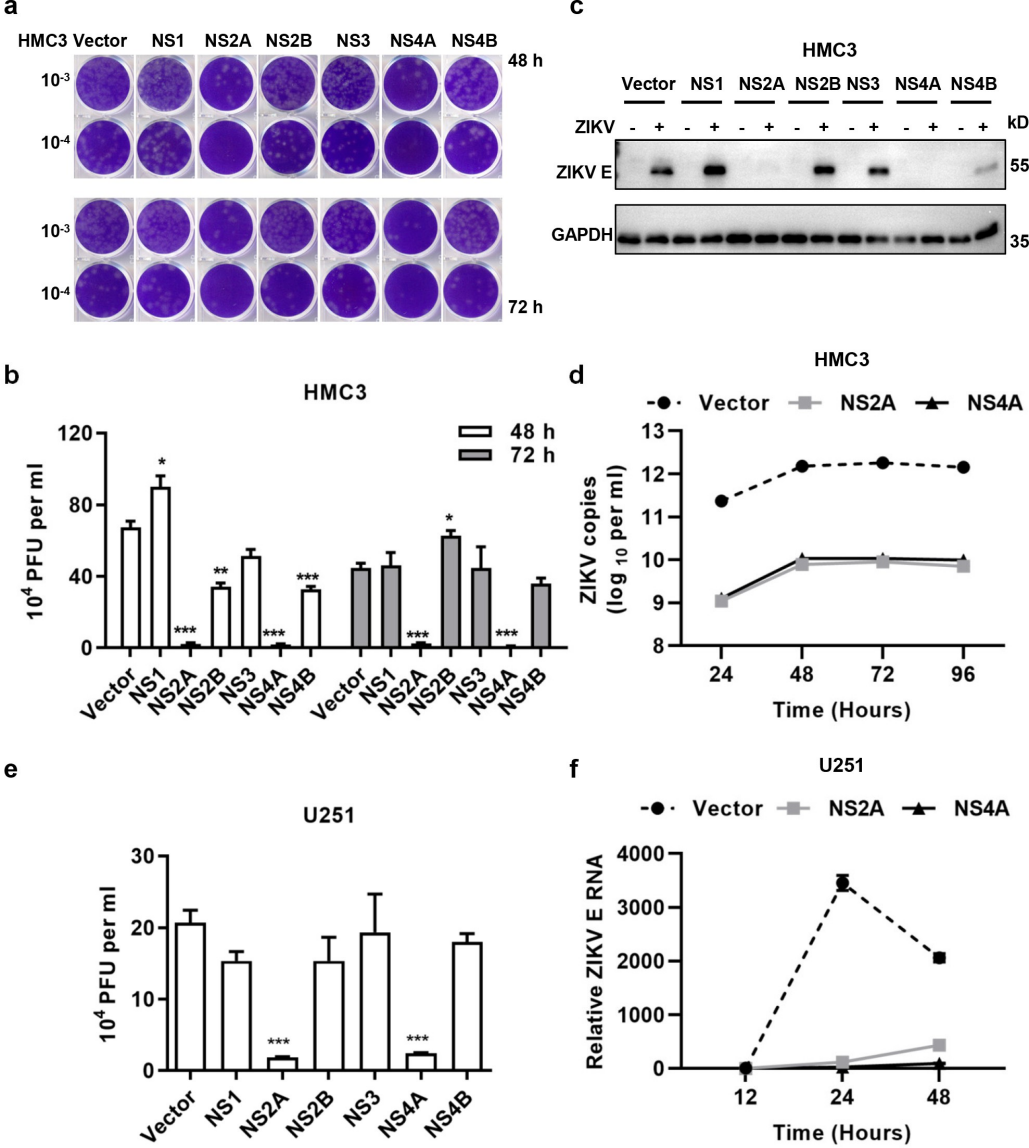
NS2A and NS4A inhibited ZIKV infection. **(a-b & e)** NS2A and NS4A reduced the production of infectious viral particles in HMC3 or U251 cells (48 h p.i.). **(c)** NS2A and NS4A inhibited the expression of ZIKV E protein in HMC3 cells. **(d)** NS2A and NS4A inhibited viral RNA replication in HMC3 cells. **(f)** NS2A and NS4A inhibited the replication of viral genome in U251 cells. HMC3 or U251 cells (expressing ZIKV NS1, NS2A, NS2B, NS3, NS4A, or NS4B) were infected by 0.1 MOI of ZIKV/SZ01 for 48 or 72 h. ZIKV titers in the culture supernatants were determined by viral plaque formation unit assay (Fig 3A, 3B and 3E) and the expression of ZIKV envelope (E) at 48 h p.i. (Fig 3C) was examined by western blot analysis. Fig 3B was the quantitative analysis of results shown in Fig 3A. ZIKV E was stained by an anti-E rabbit antibody. HMC3 or U251 cells (expressing ZIKV NS2A or NS4A) were infected by 0.1 MOI of ZIKV/SZ01 and total RNA was prepared at various time points p.i. for measuring viral RNA copies in the culture supernatants (HMC3, Fig 3D) or viral genome in cell lysates (U251, Fig 3F), determined by TaqMan or SYBR Green qRT-PCR. The differences between groups of control and NS proteins were evaluated by two-tailed Student’s t test. Data are means ± SEM of triplicate experiments; *, P< 0.05; **, P< 0.01; ***, p < 0.001.

To investigate the effect of NS protein expression on ZIKV infection, HMC3 cells expressing NS proteins were infected by 0.1 MOI of ZIKV, and the viral titers in the culture supernatants were measured by a plaque formation unit (PFU) assay. As shown in Fig 3A and 3B, the infectious viral particles in cells expressing NS2A or NS4A were significantly less than that in the control cells without NS. ZIKV E protein levels in HMC3 cells expressing NS2A or NS4A were markedly lower than that in the cells expressing other NS proteins after ZIKV infection (Fig 3C). To further verify the inhibition of ZIKV replication by NS2A and NS4A, the culture medium of ZIKV infected cells were collected at 24, 48, 72 and 96 h p.i. for measuring viral RNA copy numbers by qRT-PCR. We found that the viral RNA copies in cells expressing NS2A or NS4A were much less than those in cells without NS proteins (Fig 3D). In agreement with results from HMC3 cells, NS2A and NS4A expression in U251 cells also effectively inhibited the infection of ZIKV (Fig 3E and 3F).

### NS2A and NS4A mainly inhibited ZIKV RNA replication

**Fig 4:**
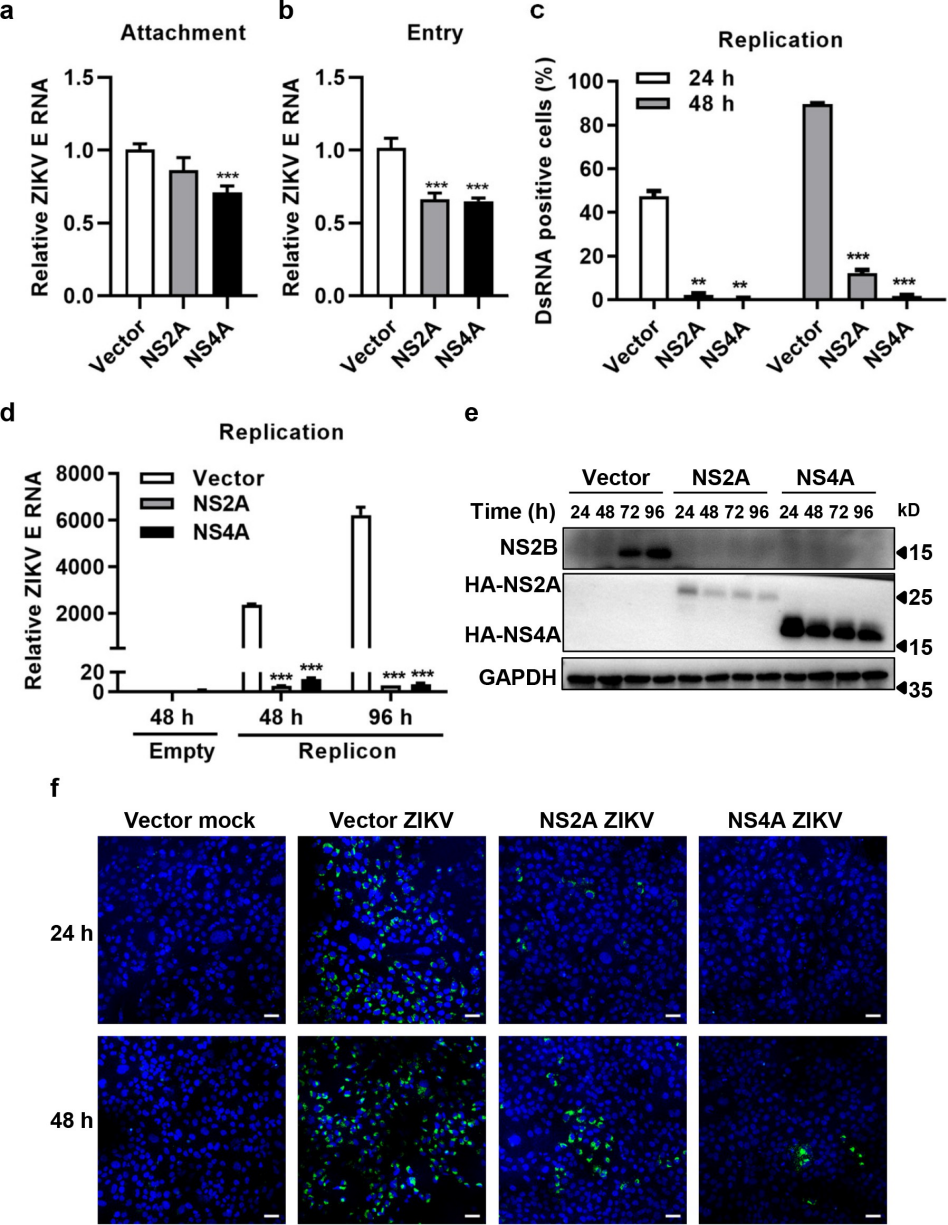
NS2A and NS4A inhibited ZIKV RNA replication. **(a)** Inhibition of ZIKV attachment to cells by NS2A and NS4A. HMC3 cells expressing NS2A or NS4A were incubated with 2 MOI of ZIKV at 4°C for 1 h, followed by washes extensively with PBS. Viral attachment was assessed by qRT-PCR. **(b)** Inhibition of ZIKV entry into cells by NS2A and NS4A. HMC3 cells expressing NS2A or NS4A were incubated with 2 MOI of ZIKV at 4°C for 1 h, followed by incubation at 37°C for another 10 min. Viral entry into cells was determined by qRT-PCR. **(c & f)** Inhibition of ZIKV dsRNA production by NS2A and NS4A. DsRNA synthesis was analyzed by IFA in HMC3 cells (expressing ZIKV NS2A or NS4A) infected with 0.1 MOI of ZIKV for 24 or 48 h (Fig 4F). DsRNA was probed by the J2 mouse monoclonal anti-dsRNA antibody (green) with cell nuclei stained by 4,6-diamidino-2-phenylindole (DAPI, blue). Scale bar, 50 μm. Four fields of each group were randomly selected for statistical analysis by Image J (Fig 4C). **(d)** Inhibition of ZIKV RNA replication by NS2A and NS4A. **(e)** Inhibition of ZIKV NS2B expression by NS2A and NS4A. HMC3 cells expressing NS2A or NS4A were transfected with RNA from ZIKV replicons. At 48 or 96 h post transfection, total RNA was prepared from the cells and ZIKV RNA copies were measured by qRT-PCR (Fig 4D). Lysates were collected to determine the expression of NS2B by western blot assay at 24, 48, 72 or 96 h.p.i. (Fig 4E). The differences between groups of controls and NS proteins were evaluated by two-tailed Student’s t test. Data are means ± SEM of at least triplicate experiments; **, P< 0.01; ***, p < 0.001.

To examine which step of viral life cycle was targeted by NS2A and NS4A, HMC3 cells expressing NS2A or NS4A were infected with ZIKV at 4°C for 1 h, followed by washing the cells to remove unbinding viruses. Total RNA was prepared from the cells to determine the viral attachment by qRT-PCR. The results showed that NS4A expression inhibited 28.9% of ZIKV attachment to HMC3 cells but NS2A expression had no such effect (Fig 4A). Subsequently, we detected the impact of NS2A and NS4A on ZIKV entry. As shown in Fig 4B, both NS2A and NS4A exhibited significant inhibitory effect on viral entry compared to the control, with the inhibition rates of 33.8% and 35.1%, respectively. At the same time, viral dsRNA was detected at 24 and 48 h p.i. As shown in Figs 4C, 4F and S5, NS2A and NS4A significantly inhibited the production of viral dsRNA. To characterize the effect of NS2A and NS4A on viral RNA replication, plasmids carrying cDNA of ZIKV full genome were transcribed into RNA *in vitro* and then the infectious RNA was electrotransfected into HMC3 cells expressing NS2A or NS4A. Total RNA were prepared from the transfected cells for viral RNA detection and cell lysates were collected to determine the expression of NS2B. NS2B is part of the replication complex and is responsible for grouping together the other components of the replication complex [37,38]. As shown in Fig 4D, NS2A and NS4A expression significantly suppressed viral RNA replication, with 177 or 795-fold lower viral copies than controls at 48 or 96 h post transfection, respectively. The expression of NS2B protein was markedly lower in the cells expressing NS2A or NS4A than that in the controls (Fig 4E). These results indicate that NS2A and NS4A mainly inhibited ZIKV RNA replication.

### NS2A and NS4A broadly inhibited viral infection

**Fig 5:**
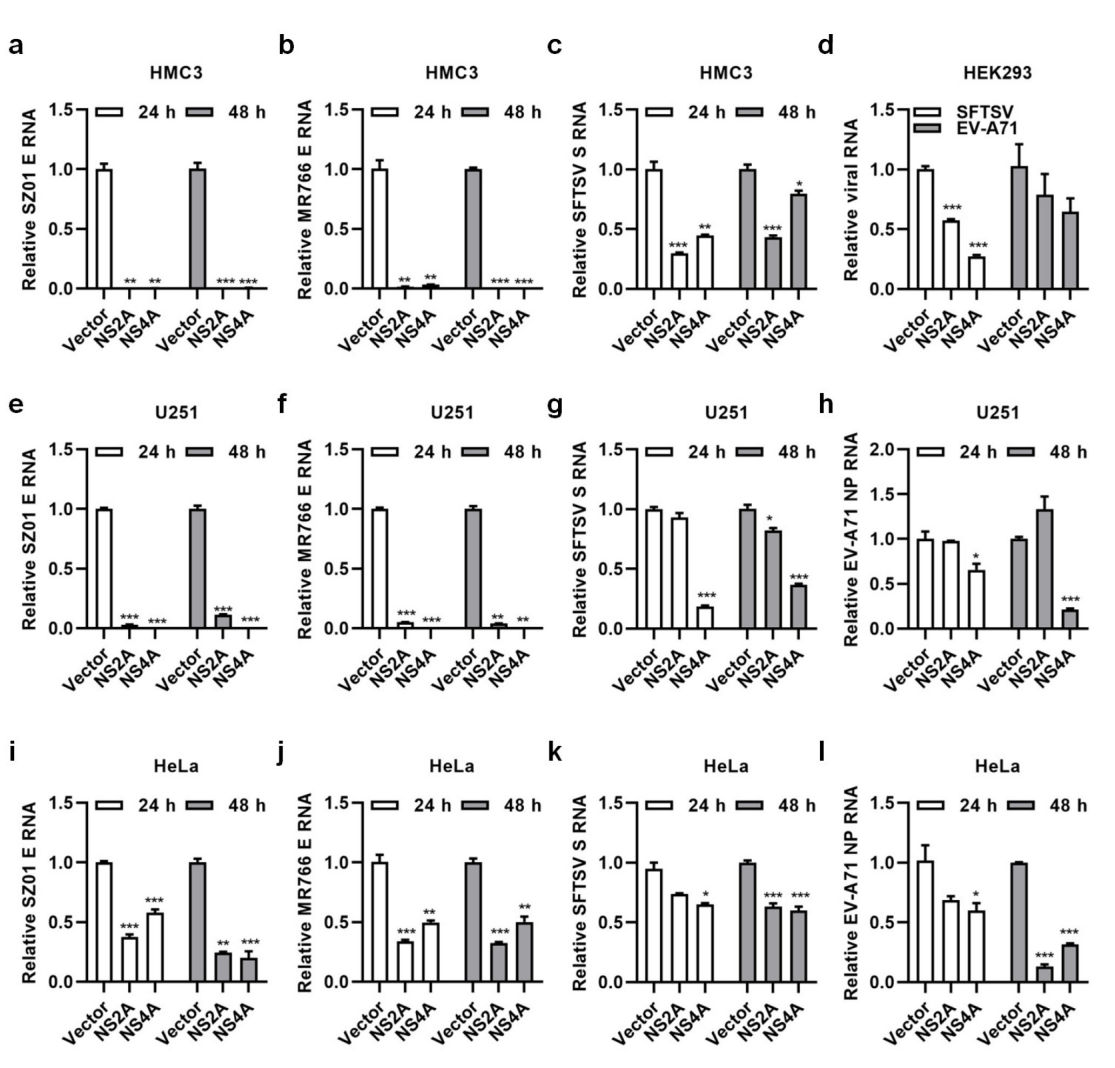
NS2A and NS4A broadly inhibited viral infection. **(a-c & e-h)** The antiviral activity of NS2A and NS4A in HMC3 or U251 cells. HMC3 or U251 cells expressing NS2A or NS4A were infected by 0.1 MOI of ZIKV/SZ01, ZIKV/MR766, SFTSV or EV-A71 for 24 and 48 h. Viral RNA copies in infected cells were determined by qRT-PCR. **(d & i-l)** Antiviral activity of NS2A and NS4A in HEK293 or HeLa cells. HEK293 or HeLa cells were transfected with plasmids expressing NS2A or NS4A for 24 h, followed by infection with ZIKV/SZ01, ZIKV/MR766, SFTSV or EV-A71 for 24 (Fig 5D) or 48 h. Viral RNA copies in infected cells were determined by qRT-PCR. The differences between groups of control and NS2A or NS4A were evaluated by two-tailed Student’s t test. Data are means ± SEM of triplicate experiments; *, P < 0.05; **, P< 0.01; ***, p < 0.001.

The above results showed NS2A and NS4A from ZIKV/SZ01 inhibited ZIKV/SZ01 infection. Here, we detected whether NS2A and NS4A inhibited the infection of ZIKV/MR766, SFTSV and EV-A71. As shown in Fig 5A and 5B and Fig 5E and 5F, expression of NS2A and NS4A could inhibit the infection of ZIKV/MR766 as effectively as did of ZIKV/SZ01. We tried two other viruses, one from *bunyaviridae*, SFTSV, and another from *piconaviridae*, EV-A71, to see if NS2A and NS4A could have any impact on them from different families. As shown in Fig 5C, 5G and 5H, replications of SFTSV and EV-A71 were also inhibited to some extents. HeLa and HEK293 cells were also tested by transfection with plasmids expressing NS2A or NS4A. As shown in Fig 5I–5L and Fig 5D, transient expression of NS2A and NS4A also could effectively inhibit the replication of ZIKV/SZ01, ZIKV/MR766, SFTSV and EV-A71 in HeLa or HEK293 cells. The above results suggested that NS2A and NS4A not only affected the process of ZIKV infection, but also might activate some host broad spectrum of antiviral responses.

### NS4A enhanced ISGs expression by activating ISGF3 pathway in HMC3 cells

**Fig 6:**
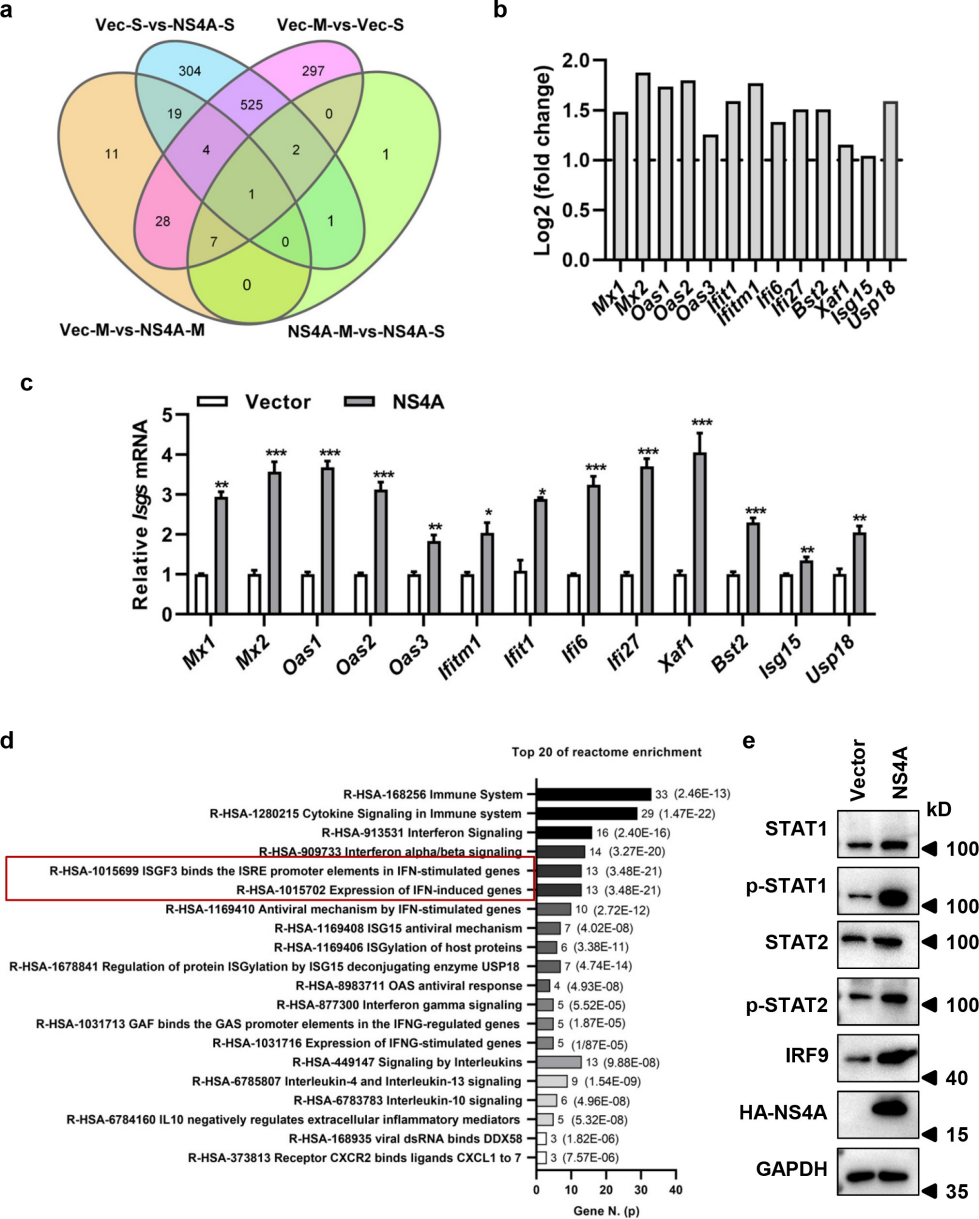
NS4A enhanced ISGs expression by activating ISGF3 pathway in HMC3 cells. **(a)** Venn Diagram of differentially expressed genes. After HMC3 cells with or without NS4A expression were infected with 0.1 MOI of ZIKV/SZ01 for 24 h, total RNA from the mock and virus infected cells were collected for RNA-Seq, followed by analyses of the differentially expressed genes. Vec-M or Vec-S, mock or ZIKV/SZ01 infected- HMC3 cells without NS4A expression; NS4A-M or NS4A-S, mock or ZIKV/SZ01 infected-group of HMC3 cells with NS4A expression. **(b)** Expression of ISGs in HMC3 cells expressing NS4A determined by RNA-Seq. **(c)** Expression of ISGs in HMC3 cells expressing NS4A verified by qRT-PCR. The differences between groups of control and NS4A were evaluated by two-tailed Student’s t test. Data are means ± SEM of triplicate experiments; *, P < 0.05; **, P< 0.01; ***, p < 0.001. **(d)** The top 20 enriched pathways of Vec-M vs NS4A-M. The pathway enrichment analysis was used to group the differentially expressed genes in HMC3 cells expressing NS4A and vector. **(e)** Detection of STAT1, p-STAT1 (Tyr701), STAT2, p-STAT2 (Tyr690) and IRF9 protein expression in the ISGF3 signaling pathway in HMC3 cells expressing NS4A by western blot analyses.

We next explore the antiviral mechanism of NS4A against ZIKV infection. HMC3 cells expressing NS4A were infected with ZIKV/SZ01 for transcriptome analysis and the differentially expressed genes in NS4A-expressing cells were determined by RNA-Seq. As shown in Fig 6A, there were more than 800 genes, which were differentially expressed between ZIKV-non-infected and infected cells (Vec-M-vs-Vec-S). Of these, 532 genes were consistent with the differentially expressed genes between the infected cells expressing or non- expressing NS4A (Vec-S-vs-NS4A-S). In contrast, there were just 12 differentially expressed genes between NS4A-expressing cells infected with or without ZIKV (NS4A-M-vs-NS4A-S), which substantiated NS4A could inhibit the replication of ZIKV in HMC3 cells. Meanwhile, there were about 70 differentially expressed genes between the cells with or without expression of NS4A (Fig 6A). Thirteen IFN-stimulated genes (ISGs), including *Mx1/2*, *Oas1/2/3*, *Ifitm1*, *Ifit1*, *Ifi6*, *Ifi27*, *Xaf1*, *Bst2*, *Isg15*, and *Usp18*, were up-regulated by NS4A expression (Fig 6B and 6C), which may be due to the activation of IFN-stimulated gene factor 3 (ISGF3) signaling pathway (Fig 6D). To verify the activation of ISGF3 signaling pathway, the expression levels of phosphorylated STAT1\STAT2 and IRF9 in HMC3 cells were analyzed by western blot assay. As shown in Fig 6E, phosphorylated STAT1\STAT2 and IRF9 increased in NS4A-expressing cells in comparison with the cells without NS4A. Taken together, these results indicated that NS4A induced the expression of ISGs through activating the ISGF3 signaling pathway.

### The ISGF3 signaling pathway was partially responsible for the antiviral effect of NS4A

**Fig 7:**
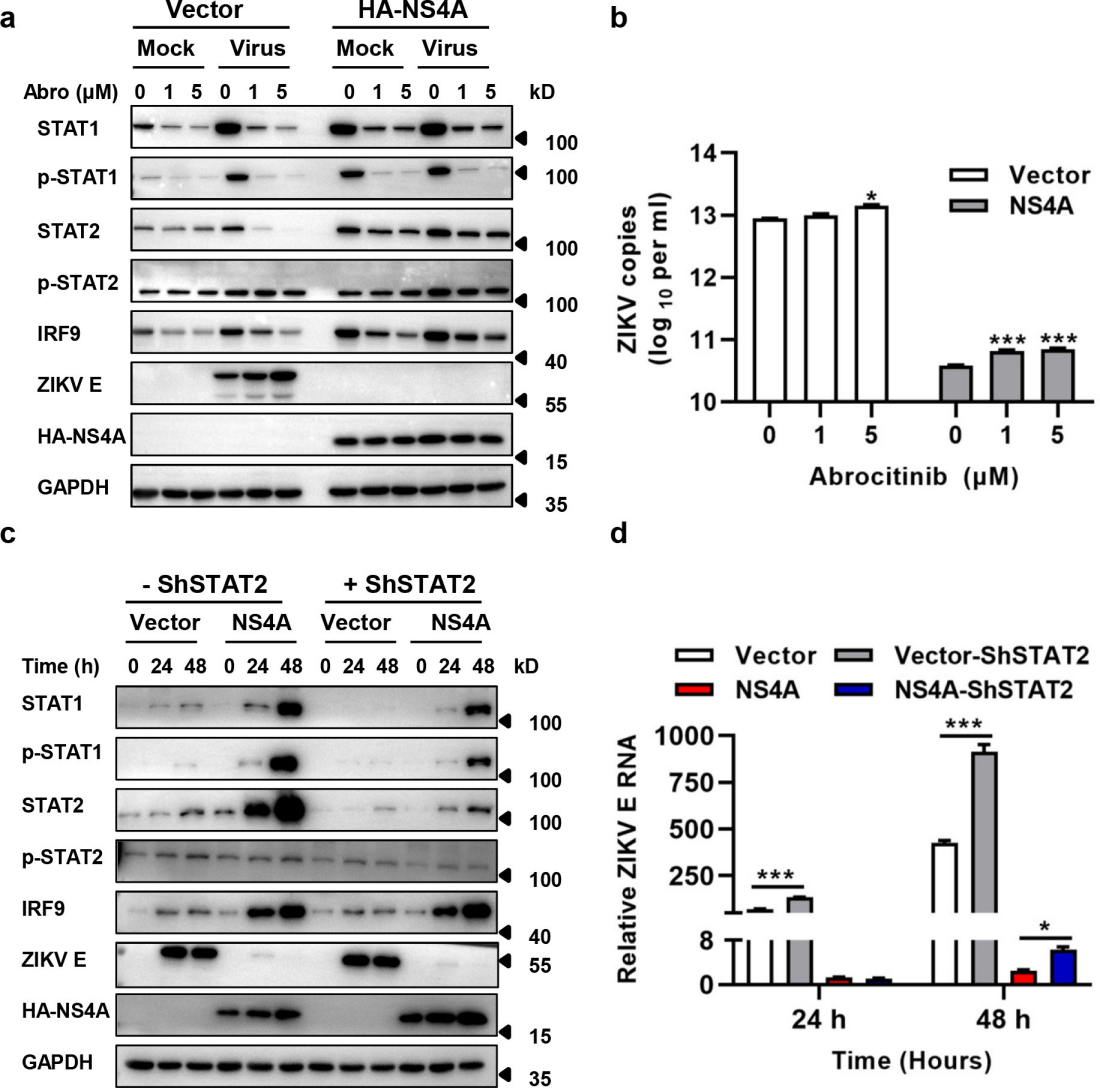
The ISGF3 signaling pathway was partially responsible for the antiviral effect of NS4A. **(a)** Effect of Abrocitinib on the expression of ZIKV E protein in HMC3 cells. **(b)** Effect of Abrocitinib on the reproduction of ZIKV in HMC3 cells. HMC3 cells expressing NS4A were infected with 0.1 MOI of ZIKV/SZ01, with (1 μM or 5 μM) or without the treatment of Abrocitinib. Protein expression of STAT1, p-STAT1 (Tyr701), STAT2, p-STAT2 (Tyr690), IRF9 and ZIKV E was detected by western blot analyses (Fig 7A) and viral RNA copies in the culture supernatants were determined by TaqMan qRT-PCR at 48 h p.i. **(c)** Effect of knockdown of STAT2 on ZIKV E protein expression in U251 cells. **(d)** Effect of knockdown of STAT2 on ZIKV E RNA replication in U251 cells. The STAT2 in U251 cells with or without NS4A expression was knocked down by shSTAT2. Subsequently, the above cells and the control cells were infected with 0.1 MOI of ZIKV /SZ01. Protein expression of STAT1, p-STAT1 (Tyr701), STAT2, p-STAT2 (Tyr690), IRF9 and ZIKV E was detected by western blot analyses (Fig 7C) and viral RNA in the cell lysates was determined by TB Green qRT-PCR (Fig 7D). The differences of ZIKV viral RNA copies between groups of control and Abrocitinib or shSTAT2 were evaluated by two-tailed Student’s t test. Data are means ± SEM of triplicate experiments; *, P< 0.05; ***, p < 0.001.

To examine the role of ISGF3 signaling pathway in the antiviral effect of NS4A, Abrocitinib (JAK1 inhibitor, also inhibition of STAT1 phosphorylation) [39,40] was used to pre-treat the cells for inhibiting the ISGF3 signaling pathway. As shown in Fig 7A Abrocitinib inhibited the phosphorylation of STAT1 and the expression of STAT1 and IRF9 effectively. In the cells without NS4A expression, ZIKV E protein obviously increased with the dose of Abrocitinib (Fig 7A). ZIKV E protein in the cells expressing NS4A was not detected in the presence of Abrocitinib, indicating that the expression of ZIKV E remains low. Though the viral copies in the culture supernatants of the cells expressing NS4A significantly increased with the dose of Abrocitinib, they were about 100 fold lower than that of cells without NS4A expression. (Fig 7B). We knocked down STAT2 with specific shRNA in U251 cells expressing NS4A to examine whether STAT2 was also involved in the antiviral activity of NS4A. As shown in Fig 7C, knockdown of STAT2 resulted in decreased expression of STAT2 and p-STAT2, but no visible increase of the ZIKV E protein expression. Although E RNA replication was significantly enhanced in the cells expressing NS4A and shSTAT2 compared to control at 48 h p.i., it was 145-fold lower than that in the cells without NS4A expression (Fig 7D). These results suggested that STAT1 and STAT2 may be less associated with the antiviral activity of NS4A.

**Fig 8:**
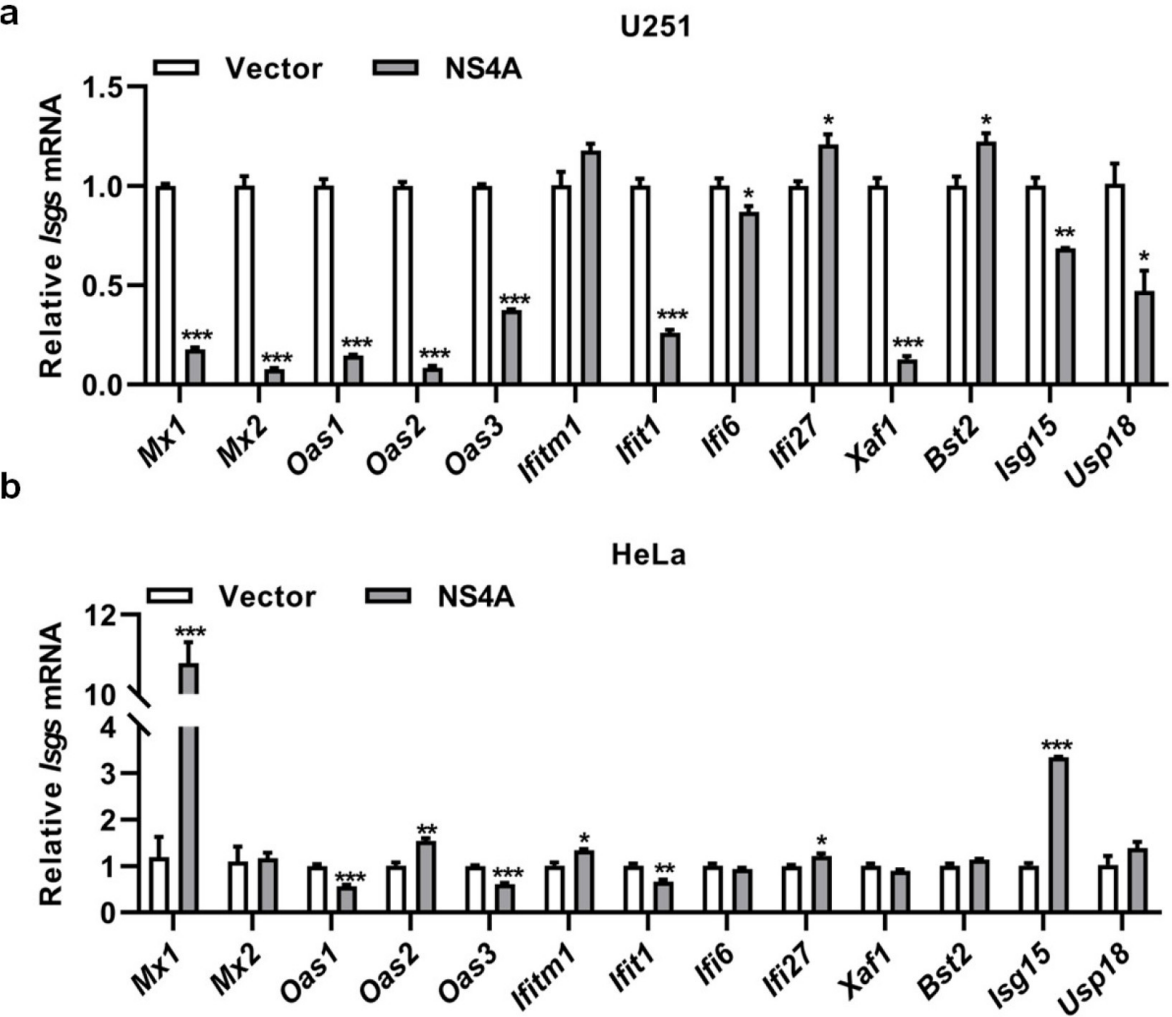
The ISGs expression induced by NS4A in U251 or HeLa cells. **(a)** U251 cells with or without NS4A expression were cultured by DMEM with 2% FBS for 24 h. Total RNA was prepared from the cells for ISG transcription by qRT-PCR. **(b)** HeLa cells were transfected with plasmids expressing NS4A or empty vector for 24 h. After the cells were cultured in DMEM with 2% FBS for 24 h, total RNA was prepared from the cells for ISG transcription by qRT-PCR. The differences between groups of control and NS4A were evaluated by two-tailed Student’s t test. Data are means ± SEM triplicate experiments; *, P< 0.05; **, P< 0.01; ***, p < 0.001.

In addition, we observed that NS4A in U251 (Fig 8A) or HeLa (Fig 8B) cells significantly induced the expression of *Ifi27* and *Bst2* or *Mx1* and *Isg15*, a different pattern from observed in HMC3 cells, probably suggesting that NS4A up-regulated some ISGs in a cell type-dependent manner. According to the above, the activation of ISGF3 signaling pathway may play a partially role in the antiviral effect of NS4A.

### Aborting NS4A protein expression rescued ZIKV RNA replication and protein expression

**Fig 9:**
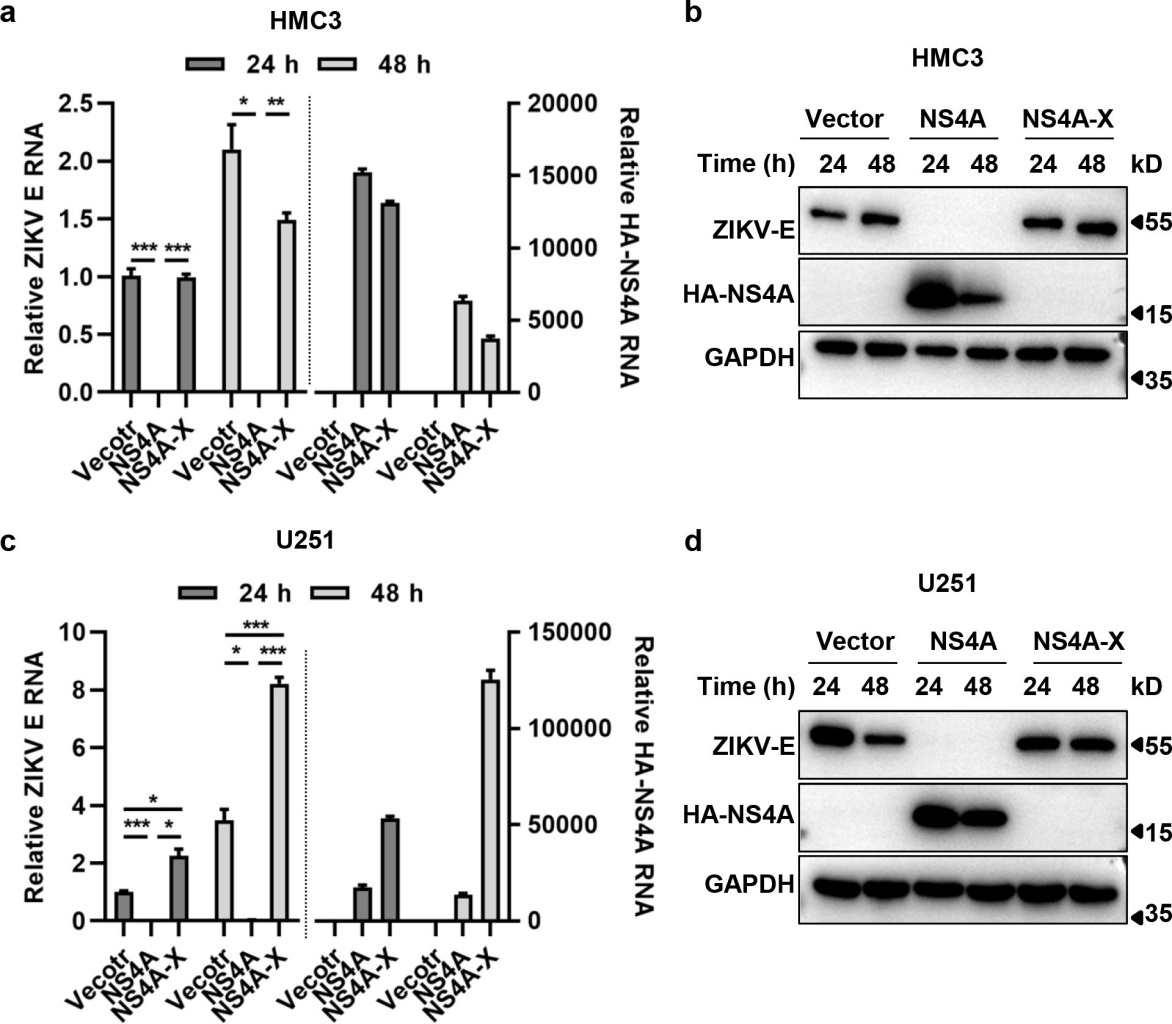
Aborting NS4A protein expression rescued ZIKV RNA replication and protein expression. **(a & c)** RNA replication of ZIKV E and HA-NS4A in HMC3 (Fig 9A) or U251 (Fig 9C) cells expressing HA-NS4A or NS4A-X. **(b & d)** Protein expression of ZIKV E and HA-NS4A in HMC3 (Fig 9B) or U251 (Fig 9D) cells expressingHA-NS4A or NS4A-X. Cells expressing HA-NS4A or NS4A-X were infected with 0.1 MOI of ZIKV/SZ01 for 24 or 48 h. Viral RNA copies and HA-NS4A in the cell lysates were determined by TB Green qRT-PCR. Protein expression of ZIKV E and HA- NS4A were detected by western blot analyses. NS4A-X: The cells were transfected with plasmids expressing HA-NS4A, which has a stop codon introduced to abort the translation of NS4A. The differences between groups of control and HA- NS4A or NS4A-X were evaluated by two-tailed Student’s t test. Data are means ± SEM triplicate experiments; *, P< 0.05; **, P< 0.01; ***, p < 0.001.

To further explore the inhibitory effect of NS4A on ZIKV replication, we generated the cell lines expressing mutant NS4A (NS4A-X) by transfecting HMC3 or U251 cells with plasmids expressing HA-NS4A, which has a stop codon introduced to abort the translation of NS4A. We specifically detected HA-NS4A RNA replication by designing the upstream primers in the HA sequence and the downstream primers in the NS4A sequence. As shown in Fig 9, even though the NS4A RNA was transcribed, NS4A protein was absent due to the termination of its translation in the transfected cells. In the absence of NS4A protein, ZIKV E RNA replication (Fig 9A and 9C) and protein expression (Fig 9B and 9D) were almost all reverted to the level of the controls in HMC3 cells, or even higher in U251 cells. The above results suggest that the expression of NS4A protein plays a major role in the inhibition of viral infection.

## Discussion

In this report, we observed a delayed appearance of ZIKV NS4A in human microglial cells and astrocytes (Fig 1). We constructed HMC3 and U251 cell lines stably expressing ZIKV NS proteins (NS1, NS2A, NS2B, NS3, NS4A, or NS4B) (S1 and S2 Figs), and analyzed the effect of NS proteins on ZIKV infection. The results showed that expression of ZIKV NS2A and NS4A activated the IFN response signaling or other unknown mechanism, which inhibited ZIKV infection through suppressing ZIKV RNA replication in HMC3 and U251 cells (Figs 2–4). We did not pursue the detection of the NS2A expression due to lack of antibodies for ZIKV NS2A.

ZIKV proteins are produced from one open reading frame into a polyprotein that is subsequently cleaved into different proteins. It is interesting how NS4A is delayed in appearance compared to other NS proteins. Even though the difference could come from various sensitivities of antibodies, we consider that the later appearance of NS4A may be due to other reasons. One would be the half-life of NS4A, which could be shorter than other NS proteins possibly due to its post-translational modifications and proteolytic processes present in the infected cells. The half-lives of NS3, NS4A, and NS5A of hepatitis C virus (HCV) were 12, 11, and 10 h, respectively, when detected by kinetic analysis in human cell lines expressing HCV open reading frame[41]. We have collected a set of preliminary data for detecting the expression of NS proteins by a parallel reaction monitoring (PRM) mass spectrometry assay. In the PRM pre-experiment with cell lysates prepared from the mixed infected cells at 24, 36, 48 h p.i., NS1, NS2A, NS2B, NS3, NS4B and NS5 were all detected, but NS4A was not (S1 Table). The low expression of NS4A could be that NS4A is prone to be degraded in the cells infected with ZIKV at certain stage of infection. ER membrane protein complex (EMC) is essential for the correct topology and stable expression of flavivirus polyproteins [42], especially multi-pass transmembrane proteins, such as NS4A. Absence of the EMC leads to degradation of NS4A [43]. Ancient ubiquitous protein 1 (AUP1), a type-III membrane protein with dual localization signals for lipid droplets (LDs) and ER, carries NS4A from lipid droplets into the autophagosome upon DENV infection [44]. Therefore, different expression trend of NS4A from other NS proteins may be related to the post-translational modifications and proteolytic processes in cells.

During infection, ZIKV persists in the male reproductive system for as long as six months [45] likely resulting in damages of testicles and fertility [46,47]. ZIKV can also persist in whole blood for up to 73.5 days and in blood cells for up to 95.4 days [48]. The establishment of ZIKV persistent infection in monocytes enhances their adhesion and transmigration [49]. ZIKV persistent infection in the placenta and brain may be related to infant microcephaly [14,50]. And glial cells, including microglia and astrocytes, may serve as viral reservoirs for ZIKV [17,22]. We hypothesize that ZIKV may reduce its replication to a level for persistent infection and our finding in this study suggest that NS4A and NS2A may somehow activate the IFN response signaling or use other mechanisms to generate a negative feedback loop which regulates viral replication and maintains a status of a persistent infection. ZIKV infection in pregnant women results in 4–6% of fetuses with microcephaly and 5–14% with congenital abnormalities [51]. NS2A and NS4A may lead to self-limiting infections through activating antiviral mechanisms, allowing infected neural cells survive till after infants are born, which may be beneficial for viral transmission.

Expression of NS2A and NS4A not only inhibited the infection of ZIKV/SZ01, ZIKV/MR766, but also suppressed SFTSV and EV-A71 replication (Fig 5), suggesting that a broad spectrum of antiviral mechanism is involved, but the activation mechanism remains elusive. ZIKV infection of microglia up-regulated STAT1/p-STAT1 and STAT2/p-STAT2 expression (Fig 6). Inhibition of p-STAT1 by Abrocitinib or knockdown of STAT2 increased viral replication (Fig 7), which is consistent with Martinez Viedma’s findings [13]-ZIKV infection upregulated the expression of STAT2 in microglia cells, and knockdown of STAT2 elevated ZIKV replication. However, Abrocitinib or knockdown of STAT2 did not completely remove the antiviral effect of NS4A. Aborting the expression of NS4A protein eliminated its antiviral activity effectively (Fig 9), indicating that it is the NS4A protein, not RNA, that stimulates the antiviral activities and affects the viral replication. Thus, NS4A may function as a unique type of PAMP to stimulate IFN response signaling, or through other mechanisms including its impact on viral entry. Annette von dem Bussche reported that NS2 of HCV triggered endoplasmic reticulum stress and suppressed its own viral replication [52]. The expression of NS2A and NS4A is mainly in ER [53], which is likely to activate ER stress and thus inhibit RNA transcription and protein expression. Host factors interacting with NS2A and NS4A may also participate in the mechanism, which warrants further investigations.

Numerous studies have shown that ISGs are effective inhibitors of viral infections. In this report we showed that NS4A significantly induced the expression of 13 ISGs, including MX1/2, OAS1/2/3, IFITM1, IFIT1, IFI6, IFI27, XAF1, BST2, ISG15, and USP18 (Fig 6B and 6C) in microglial cells, but NS4A only up-regulated the expression of IFI27 and BST2 in U251 or MX1 and ISG15 in HeLa cells (Fig 8). IFI27 significantly inhibited the replication of HCV [54], while BST2 inhibited the release of DENV [55]. Both HCV and DENV are members of the family *Flaviviridae*. Knockdown of MX1 significantly inhibited anti-ZIKV activity by IFN-λ in a human trophoblast line (JEG3) [56]. Silencing of ISG15 enhanced ZIKV infectivity, while supplementation with recombinant ISG15 inhibited ZIKV infection of primary human corneal epithelial cells [57]. We speculate that IFI27, BST2, MX1 and ISG15 may play an important role in the suppression of ZIKV and other viral infections rendered by NS4A.

NS4A may up-regulate ISGs in a cell type-dependent manner. Microglial cells have a unique RNA expression profile and even are heterogeneous in different locations in the brain [58]. ZIKV induces type I IFN in primary human placental macrophages (Hofbauer cells) [59] and type III IFN in human placental trophoblasts [60]. OAS2, ISG15, and MX1 are expressed in human skin cells [61], while ISG15, HERC5, and USP18 are expressed in brain microvascular endothelial cells [62]. ZIKV inhibits type-I IFN production and downstream signaling in A549 cells [26]. It is imaginable that ZIKV infection induces different host responses in different tissues and cells.

In conclusion, we observed that the expression of ZIKV NS2A and NS4A inhibited ZIKV infection in microglia and astrocytes probably through activation of antiviral mechanisms for suppression of viral RNA replication. NS4A enhanced ISGs expression by activating ISGF3 signaling pathway, and blocking the ISGF3 pathway could partially repress the antiviral activity of NS4A. Aborting the expression of NS4A protein eliminates its antiviral activity effectively. NS4A protein occurred later than NS2B, NS3 and NS5 during ZIKV infection. We hypothesize that ZIKV NS4A may regulate viral replication by inducing an innate immune response, working as a PAMP, or functioning with other mechanisms, to help maintain a status of viral persistent infection. Our study will be helpful in further characterizing and understanding viral neuropathogenesis in ZIKV infection.

## Supporting information

S1 Fig ZIKV infected and replicated in HMC3 or U251 cells. **(a & d)** ZIKV/SZ01 infection of HMC3 or U251 cells was detected by IFA. ZIKV E was stained by an anti-E mouse antibody 4G2 (green); cell nuclei were stained by 4,6-diamidino-2-phenylindole (DAPI, blue). Scale bar, 50 μm. **(b)** Cell death induced by ZIKV infection in HMC3 cells was analyzed by flow cytometry and Annexin V/PI apoptosis Kit. **(c & f)** ZIKV replication in HMC3 or U251 cells was determined by a viral plaque formation unit assay. **(e)** Cytopathic effect was observed under a light microscope after ZIKV infection in U251 cells. Scale bar, 50 μm.
(TIF)

S2 Fig Generation of HMC3 cells expressing ZIKV NS proteins by a lentivirus vector. **(a)** The expression of ZIKV NS proteins in HMC3 cells was analyzed by IFA. HA-tagged ZIKV NS proteins were detected by staining with anti-HA rabbit antibodies (green); cell nuclei were stained by DAPI (blue). Scale bar, 20 μm. (**b**) The expression of ZIKV NS proteins in HMC3 cells was analyzed by western blot. HA-tagged ZIKV NS proteins were detected by anti-HA rabbit antibodies. The red box indicates the targeted band.
(TIF)

S3 Fig Generation of U251 cells expressing ZIKV NS proteins by a lentivirus vector. (**a**) The expression of ZIKV NS proteins in U251 cells was analyzed by IFA. HA-tagged ZIKV NS proteins were detected by anti-HA rabbit antibodies (green); Cell nuclei were stained by DAPI (blue). Scale bar, 20 μm. (**b**) The expression of ZIKV NS proteins in U251 cells was analyzed by western blot. HA-tagged ZIKV NS proteins were detected by anti-HA rabbit antibodies. The red box indicates the targeted band.
(TIF)

S4 Fig NS2A and NS4A protected HMC3 and U251 cells against ZIKV infection. **(a-b)** HMC3 or U251 cell death was determined by flow cytometry and Annexin V/PI apoptosis kit at 48 (U251) or 72 h (HMC3) post ZIKV/SZ01 (0.1 MOI) infection.
(TIF)

S5 Fig Inhibition of ZIKV dsRNA production by NS2A and NS4A. DsRNA production was analyzed by IFA in HMC3 cells infected with 0.1 MOI of ZIKV for 48 h. DsRNA was probed by the J2 mouse monoclonal anti-dsRNA antibody (green) with cell nuclei stained by 4,6-diamidino-2-phenylindole (DAPI, blue). Magnification: 60X.
(TIF)

S1 Table Skyline analysis of the data of parallel reaction monitoring (PRM) mass spectrometry assay for target peptides of ZIKV non-structural proteins. (XLSX)
