# Supplementary figures and images for: Intrinsic features of Zika Virus non-structural proteins NS2A and NS4A in the regulation of viral replication
Source: PLoS Negl Trop Dis. 2022 May 6;16(5):e0010366. doi: 10.1371/journal.pntd.0010366 (PMC9075646; doi:10.1371/journal.pntd.0010366)

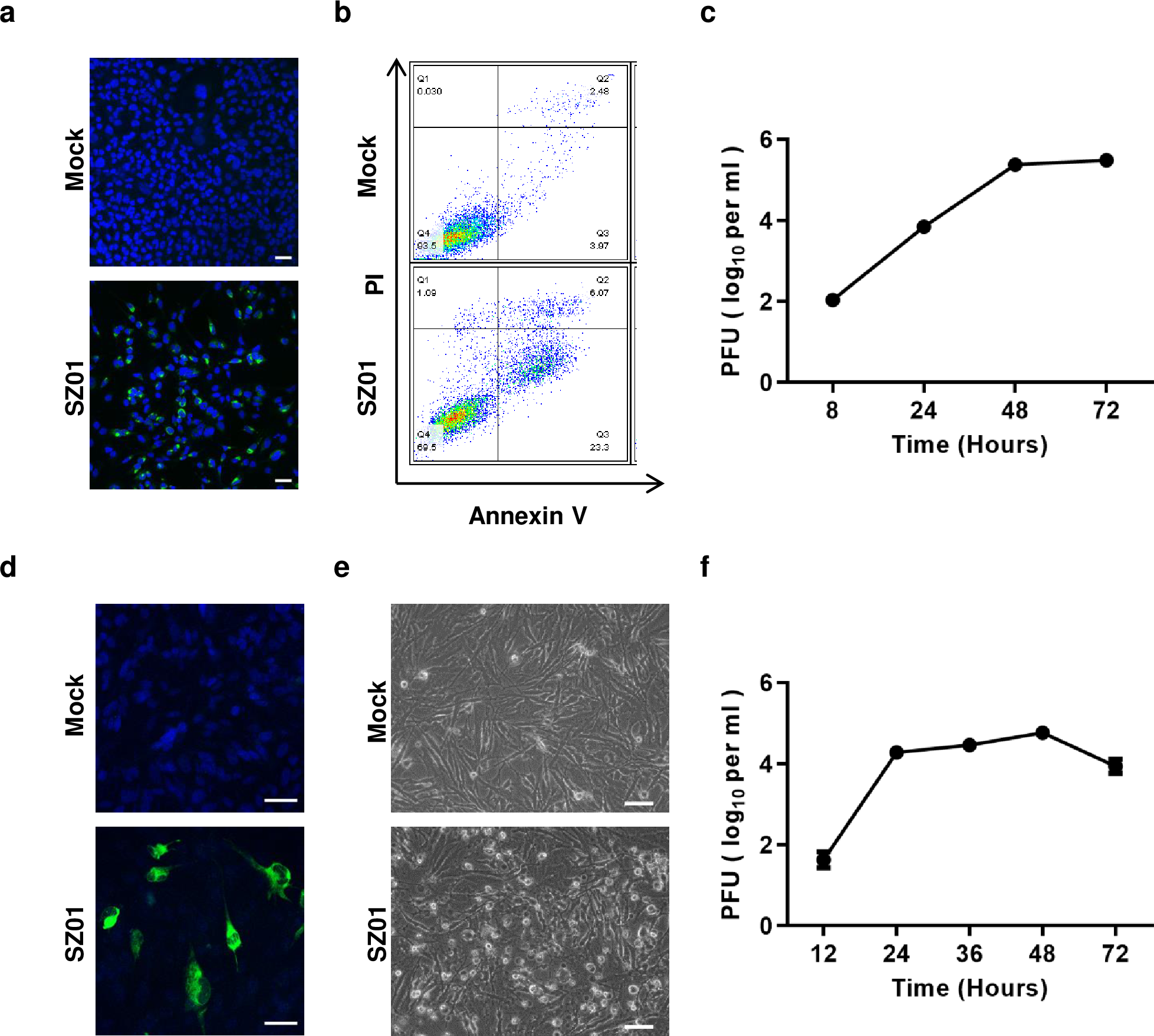

Supplement: S1 Fig — (a & d) ZIKV/SZ01 infection of HMC3 or U251 cells was detected by IFA. ZIKV E was stained by an anti-E mouse antibody 4G2 (green); cell nuclei were stained by 4,6-diamidino-2-phenylindole (DAPI, blue). Scale bar, 50 μm. (b) Cell death induced by ZIKV infection in HMC3 cells was analyzed by flow cytometry and Annexin V/PI apoptosis Kit. (c & f) ZIKV replication in HMC3 or U251 cells was determined by a viral plaque formation unit assay. (e) Cytopathic effect was observed under a light microscope after ZIKV infection in U251 cells. Scale bar, 50 μm. (TIF) [file pntd.0010366.s001.tif]

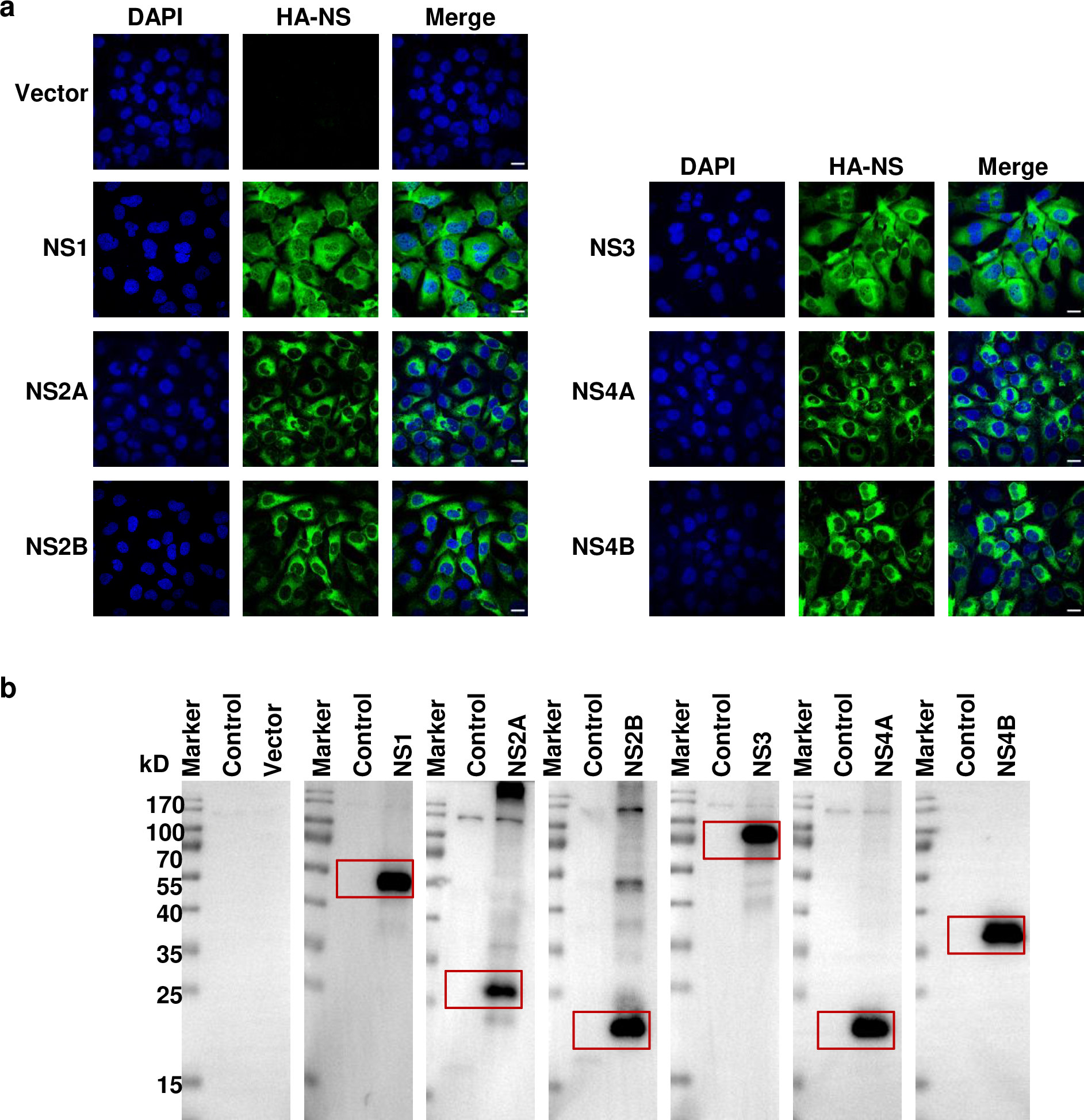

Supplement: S2 Fig — (a) The expression of ZIKV NS proteins in HMC3 cells was analyzed by IFA. HA-tagged ZIKV NS proteins were detected by staining with anti-HA rabbit antibodies (green); cell nuclei were stained by DAPI (blue). Scale bar, 20 μm. (b) The expression of ZIKV NS proteins in HMC3 cells was analyzed by western blot. HA-tagged ZIKV NS proteins were detected by anti-HA rabbit antibodies. The red box indicates the targeted band. (TIF) [file pntd.0010366.s002.tif]

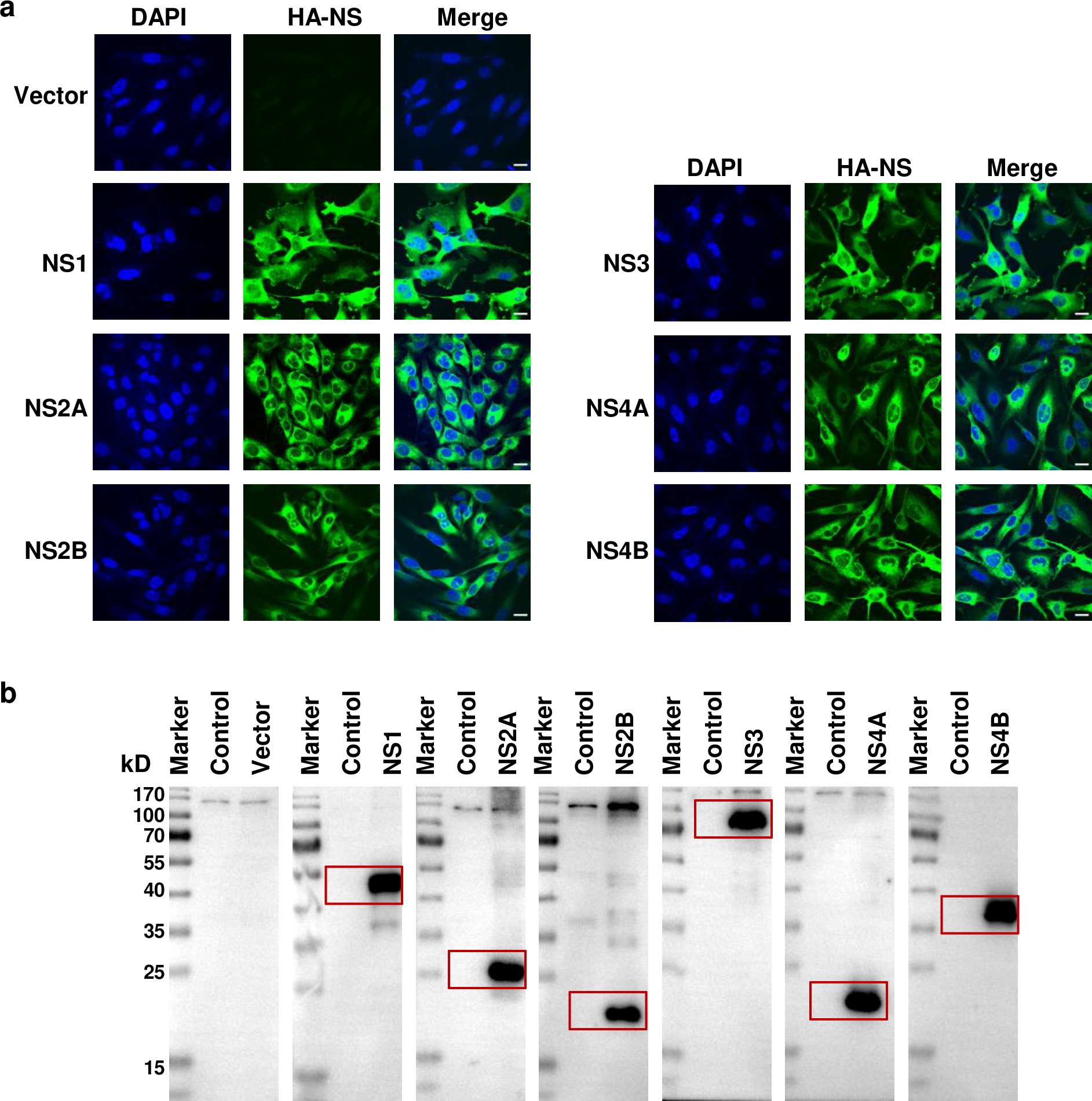

Supplement: S3 Fig — (a) The expression of ZIKV NS proteins in U251 cells was analyzed by IFA. HA-tagged ZIKV NS proteins were detected by anti-HA rabbit antibodies (green); Cell nuclei were stained by DAPI (blue). Scale bar, 20 μm. (b) The expression of ZIKV NS proteins in U251 cells was analyzed by western blot. HA-tagged ZIKV NS proteins were detected by anti-HA rabbit antibodies. The red box indicates the targeted band. (TIF) [file pntd.0010366.s003.tif]

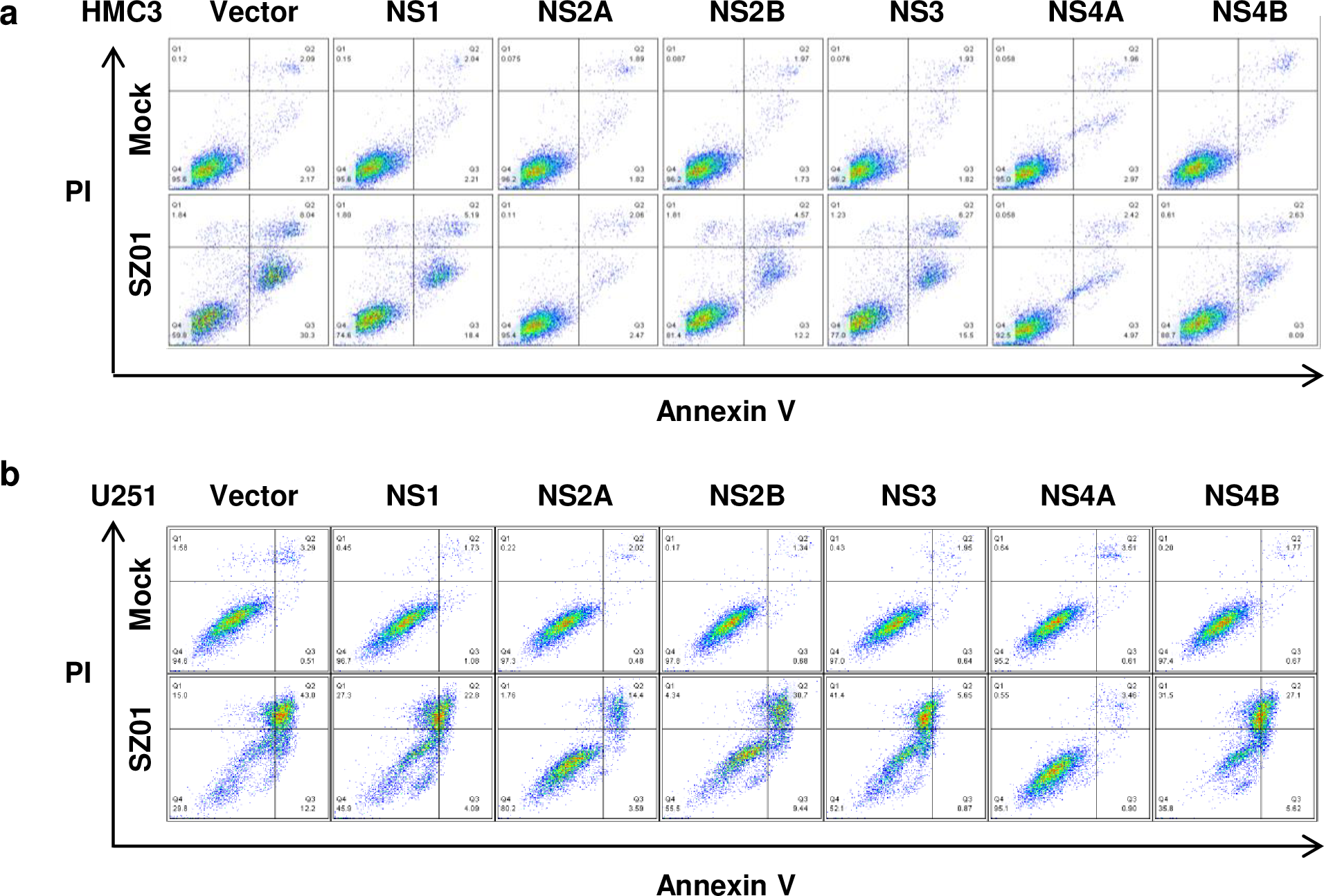

Supplement: S4 Fig — (a-b) HMC3 or U251 cell death was determined by flow cytometry and Annexin V/PI apoptosis kit at 48 (U251) or 72 h (HMC3) post ZIKV/SZ01 (0.1 MOI) infection. (TIF) [file pntd.0010366.s004.tif]

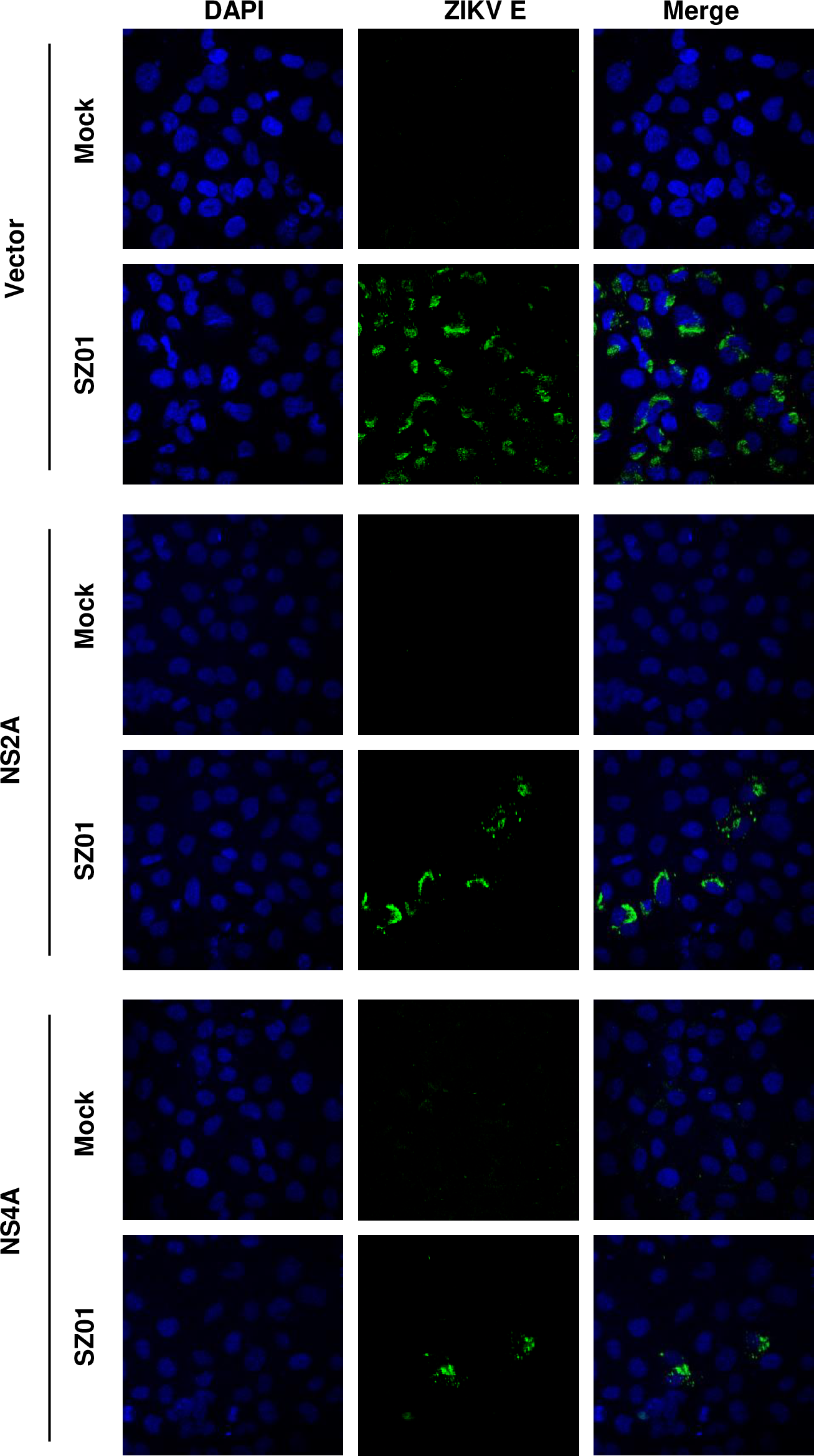

Supplement: S5 Fig — DsRNA production was analyzed by IFA in HMC3 cells infected with 0.1 MOI of ZIKV for 48 h. DsRNA was probed by the J2 mouse monoclonal anti-dsRNA antibody (green) with cell nuclei stained by 4,6-diamidino-2-phenylindole (DAPI, blue). Magnification: 60X. (TIF) [file pntd.0010366.s005.tif]
